# Roles of the Maternal and Child Health Handbook and Other Home-Based Records on Newborn and Child Health: A Systematic Review

**DOI:** 10.3390/ijerph18147463

**Published:** 2021-07-13

**Authors:** Rogie Royce Carandang, Jennifer Lisa Sakamoto, Mika Kondo Kunieda, Akira Shibanuma, Ekaterina Yarotskaya, Milana Basargina, Masamine Jimba

**Affiliations:** 1 Department of Community and Global Health, Graduate School of Medicine, The University of Tokyo, Tokyo 113-0033, Japan; jlsakamoto@m.u-tokyo.ac.jp (J.L.S.); mika.kunieda@gmail.com (M.K.K.); shibanuma@m.u-tokyo.ac.jp (A.S.); mjimba@m.u-tokyo.ac.jp (M.J.); 2 Faculty of Policy Management, Keio University, Kanagawa 252-0882, Japan; 3 National Medical Research Center for Obstetrics, Gynecology and Perinatology Named after Academician V.I. Kulakov of the Ministry of Health of the Russian Federation, 117-997 Moscow, Russia; inter_otdel@mail.ru; 4 Department of Neonatal Pathology, National Medical Research Center for Children’s Health, 119-991 Moscow, Russia; basargina.ma@nczd.ru

**Keywords:** home-based records, MCH handbook, newborn health, child health, systematic review

## Abstract

Home-based records are paper or electronic records retained and used by mothers or caregivers to document the health services received for maternal, newborn, and child health. Little has been studied about the roles of these records on newborn and child health outcomes. Hence, we collated and summarized evidence concerning the roles of home-based records in improving newborn and child health. We conducted a systematic search in several databases: MEDLINE, Web of Science, CINAHL, PsycINFO, PsycARTICLES, Academic Search Complete, SocINDEX, CENTRAL, DARE, NHS EED, HTA, J-STAGE, Ichushi, and gray literature. We included original research articles of all study designs published in English or Japanese until January 2020. Owing to heterogeneity across the outcomes of included studies, we conducted a narrative synthesis. We included 55 studies (23 in Japanese) among 14,017 identified articles. We identified the following roles of home-based records on newborn and child health: promoted newborn/childcare seeking, improved knowledge and practices of newborn/childcare, encouraged home care for childhood illnesses, reduced child mortality and morbidity, and facilitated continuum of care. We observed a mixed effect on age-appropriate immunization (e.g., DTP3 completion) and no effect on the practice of immediate breastfeeding and prevention of perinatal mortality and morbidity. The findings highlighted the effectiveness and usefulness of home-based records to improve newborn and child health outcomes. However, only a few studies were available for each outcome category, limiting the certainty of evidence provided in this review. Therefore, we recommend further studies to explore the benefits of home-based records on improving newborn and child health.

## 1. Introduction

Home-based records have been used in over 163 countries worldwide to improve maternal, newborn, and child health (MNCH) [1]. The records varied greatly in design and content across countries and regions. Home-based records are paper or electronic health records retained and used by mothers or caregivers in the household to document the health services received for MNCH [1]. They ranged from maternity case notes or vaccination-only cards to child health books or integrated maternal and child health handbooks. The record is intended to be integrated into the routine health information system and complement records maintained by health facilities [1].

Traditionally, MNCH programs have developed their own program-specific, home-based records. In many countries, the maternal health card continues to be a stand-alone record independent from child records [2]. The difference in target groups has prompted policy debates on whether home-based records should be developed and distributed separately for the mother and child [2]. The lack of integration and having no standardized format and design of home-based records might impact health reporting, health outcomes, scaling up, and evaluation between health systems worldwide [3].

The first integrated home-based record was made in Japan in 1948. The Ministry of Health, Labor and Welfare of Japan introduced the Maternal and Child Health (MCH) handbook to improve the health of both mothers and children [4]. The initial MCH handbook covered the entire spectrum of pregnancy, childbirth, postpartum, and newborn care, through childhood until six years old. In 1991, municipalities and towns/villages in Japan started distributing the MCH handbook following decentralization [4]. Local governments could add more information from the national version (consisted of 48 pages) based on their local needs [4]. Due to Japan’s success in reducing its infant mortality rate, the MCH handbook was adapted worldwide. To date, more than 50 countries used the integrated MCH handbook [4] and is particularly useful when access to health services is restricted [5].

The full integration has several advantages. It includes greater assurance of a continuum of MCH care [6,7,8,9] and sizable cost savings in the record’s operation (e.g., production and distribution) [10]. Healthcare providers can enter medical records into the handbooks and share their knowledge and information on maternal health and child growth [4]. Furthermore, parents can obtain appropriate information easily and rely on the continuous guidance of the handbook from pregnancy to childhood [4]. The MCH handbook can be a valuable tool for promoting a life-course approach to healthcare. Such an approach might help achieve Sustainable Development Goal (SDG) 3—to “ensure healthy lives and promote well-being for all at all ages” [11].

Despite substantial progress over the past two decades, in 2019 alone, the World Health Organization (WHO) reported that approximately 7.4 million children, adolescents, and youth mainly died from preventable or treatable causes [12]. Of these deaths, children under five account for 5.2 million, and the leading causes of their death include preterm birth complications, pneumonia, neonatal sepsis, diarrhea, and malaria [13]. These deaths can be prevented or treated with access to affordable and straightforward interventions, such as immunization, adequate nutrition, safe water, and quality care by a trained healthcare provider [13]. While proven and cost-effective interventions are available to reduce deaths for children under five [14,15,16,17,18], review studies that explored the roles of home-based records in promoting healthcare service delivery among this population are limited.

One study systematically reviewed the effectiveness of home-based records on MNCH [3], which was used as a basis for the WHO’s recommendations concerning home-based records. However, this review article did not comprehensively examine the roles of home-based records on newborn and child health. As defined in the WHO guideline [1], newborn and child health outcomes include newborn/child morbidity and mortality, care seeking, care practices, and health services utilization. Magwood et al. [3] included only two studies for newborn health and seven studies for child health outcomes. They reported no significant effects on newborn health outcomes (e.g., neonatal death or stillbirths, immediate breastfeeding). For child health outcomes, they reported that home-based records might positively impact immunization rates, growth, and development. However, they covered only original research articles with randomized controlled study designs and that were written in English. They might have missed valuable information derived from observational studies. Observational studies may provide more evidence on the roles of home-based records on newborn and child health. They provide information on actual adherence to home-based records, bringing evidence on real-life outcomes of records. In addition, since Japan is a proponent of the MCH handbook, it would be worthy of including Japanese articles in the analysis. By doing so, we could capture more evidence on the roles of home-based records on newborn and child health. Thus, this systematic review collated and summarized evidence from all study designs, available in English and Japanese, to assess the roles of home-based records on newborn and child health outcomes.

## 2. Methods

### 2.1. Patient and Public Involvement Statement

Patients and the public were not involved in the design or planning of this study.

### 2.2. Review Protocol

**Figure 1:**
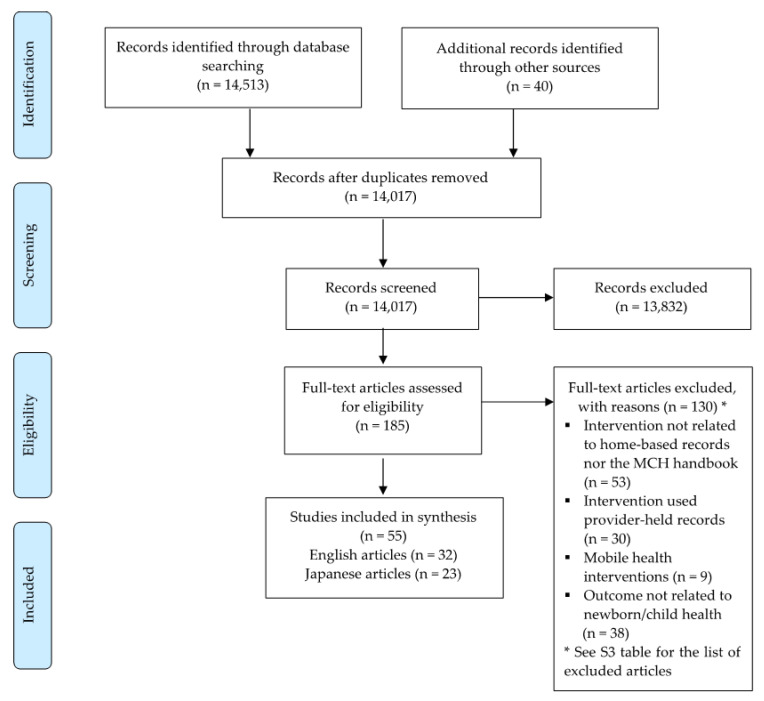
PRISMA flow diagram of the screening process.

This systematic review initially followed the Preferred Reporting Items for Systematic Review and Meta-Analyses (PRISMA) reporting guidelines [19] (Figure 1). We developed a review protocol registered on PROSPERO (no. CRD42020166545; Text S1).

### 2.3. Selection Criteria

#### 2.3.1. Population

We reviewed studies involving parents, fathers, mothers, and caregivers of newborns and children. Pregnant and postpartum women were included in the study. We considered all health settings, including the community.

#### 2.3.2. Intervention

The intervention of interest was the MCH handbook and other home-based records, available in hard copy and kept or managed by parents or caregivers. Home-based records ranged from maternity case notes or vaccination-only cards to child health books or integrated maternal and child health books. We excluded patient diaries, provider-held records (e.g., electronic medical records), and mobile health interventions that involved using applications (apps) and text messages. Patient web-based summaries of their appointments were also excluded, as they fall under electronic medical records.

#### 2.3.3. Comparison

The comparator was no record, conventional information, or standard care given to parents or caregivers before or following childbirth. Studies on the intervention without reference to a comparison group were also included in this review.

#### 2.3.4. Outcome

We included newborn and child health outcomes following the WHO guideline [1], such as newborn/child morbidity and mortality, care seeking, care practices, and health services utilization. We included care knowledge because of its importance in the chain of outcomes. Knowledge leads to improved practices and care seeking, leading to improved health and well-being. We also provided specific outcomes under each aforementioned outcome category as reported in both English and Japanese articles.

#### 2.3.5. Types of Studies

We included original research articles in English and Japanese across all study designs such as randomized controlled trials (RCT), quasi-experimental, cohort, cross-sectional, and other comparative studies, as well as case studies and evaluation reports. We excluded letters, editorials, reviews, conference abstracts, and books.

### 2.4. Search Strategy

The first two authors (R.R.C. and J.L.S.) developed the search strategy. Two of us searched the following electronic databases for English articles: MEDLINE, Web of Science, CINAHL, PsycINFO, PsycARTICLES, Academic Search Complete, SocINDEX, Cochrane Central Register of Controlled Trials (CENTRAL), The Database of Abstracts of Reviews of Effects (DARE), NHS Economic Evaluation Database (NHS EED), and the Health Technology Assessment (HTA) database.

We also conducted our search for Japanese databases. J.L.S. and M.K.K. conducted the evidence search in Japanese using J-STAGE (https://www.jstage.jst.go.jp/) (accessed on 31 January 2020) and Igaku-chuo-zasshi (Ichushi; https://search.jamas.or.jp/) (accessed on 31 January 2020). Both J-STAGE and Ichushi cite over 300,000 articles per year from 2500 Japanese biomedical journals. We used a combination of Medical Subject Headings (MeSH) terms and keywords (Text S2) and applied no date restriction. We also searched gray literature from the US Center for Disease Control and Prevention, the European Centre for Disease Prevention and Control, United Nations Children’s Fund, Japan International Cooperation Agency, and WHO databases. We included all original articles in the English and Japanese language published on or before January 2020. Moreover, we hand-searched the reference list of articles selected for full texts. To facilitate the study selection process, we uploaded the search records to a reference-managing software package (Endnote X9).

### 2.5. Evidence Retrieval

The first three authors (R.R.C., J.L.S. and M.K.K.) retrieved 14,513 articles from all the databases, and an additional 40 articles were hand-searched. Of these, 823 were Japanese articles. After removing duplicates, we obtained 14,017 articles. Then, two researchers independently performed the eligibility assessment in a blinded standardized manner. R.R.C. and J.L.S. conducted the eligibility assessment of the English articles, while M.K.K. and J.L.S. did so for the Japanese articles. Three of us resolved any disagreements through discussion until a consensus was reached or, if required, by consulting a fourth author (A.S. or M.J.) for the final decision. Upon initial screening based on titles and abstracts, we excluded 13,832 articles. This process led to the retention of 185 articles, and we screened their full texts.

R.R.C. and J.L.S. obtained full-text articles from the University of Tokyo Library system, while M.K.K. did so from the Keio University KOSMOS system and the National Diet Library Online. We further removed 130 articles (Table S1) from the list for the following reasons: neither related to home-based records nor the MCH handbook (*n* = 53), provider-held records (e.g., electronic medical records) (*n* = 30), mobile health interventions (*n* = 9), and not related to newborn/child health outcomes (*n* = 38). Overall, we included 55 articles (23 articles in Japanese) in the narrative synthesis. Figure 1 shows the PRISMA flow diagram of the screening process.

### 2.6. Data Extraction

Three authors (R.R.C., J.L.S., and M.K.K.) created a review library of included studies as PDF files using Endnote referencing software. We extracted the data independently into Microsoft Excel following the population, intervention, comparison, and outcome (PICO) format. Extracted data were title, citation (author, publication year, source), study location, objectives, study design, study setting, study population, sample size, types of home-based records, comparison group, and reported outcomes (Table S4). We discussed strategies and data presentation among researchers throughout the process of data extraction.

### 2.7. Risk of Bias and Quality of Evidence

Two authors (English articles: R.R.C. and J.L.S.; Japanese articles: M.K.K. and J.L.S.) independently assessed the risk of bias for the included studies. We used the revised Cochrane risk of bias tool (RoB 2), developed by Cochrane collaboration for RCTs [20]. We evaluated the overall risk of bias for RCTs based on the following criteria: bias arising from the randomization process, bias due to deviations from the intended intervention, bias due to missing outcome data, bias in the measurement of the outcome, and bias in the selection of the reported results [20]. We used a series of questions to elicit information about the features of RCTs that are relevant to the risk of bias. We followed the algorithm results and judged the RCTs as low, some concerns, or high risk of bias.

For non-RCTs, we used the following tools: ROBINS-I for quasi-experimental studies [21], NIH quality assessment tool for observational cohort and cross-sectional studies [22], critical appraisal skills program checklist for qualitative studies [23], and mixed methods appraisal tool for mixed-method studies [24]. We settled any disagreements through discussion and reached a consensus among the reviewers. We assessed the certainty of the evidence using the grading of recommendations assessment, development, and evaluation (GRADE) approach [25].

### 2.8. Data Analysis

All authors took part in the data analysis. We conducted a narrative synthesis for two reasons: heterogeneity across outcomes of included studies and the lack of pooled data for a meta-analysis. For this, we followed the synthesis without meta-analysis (SWiM) reporting guidelines [26] instead of the initially planned PRISMA guidelines. We conducted a detailed examination of the numeric and textual summary findings, and conclusions were reached in each study for the effects of the intervention—MCH handbook and other home-based records. For instance, we considered an outcome to have a “positive effect” if the home-based record showed a statistically significant effect (e.g., performed better than a comparator or control) and narrative findings indicated positive results (i.e., benefits of using home-based records). We coded an outcome as having a “mixed effect” when it showed some evidence of the usefulness of the record but not effectiveness per se. We classified an outcome as having “no effect” when there was no significant effect, and narrative findings indicated negative results (i.e., performed worse than the comparator). Of the 55 studies synthesized, 10 were RCTs, and the rest of the studies were observational. Studies were grouped for synthesis according to the following research questions:

1. Should home-based records (intervention) compared to no use of any home-based records (control) be used for improving newborn/child health outcomes?
2. Should a different type of home-based record (intervention) compared to a standard home-based record (control) be used for improving newborn/child health outcomes?

We presented quantitative effect sizes (those that have not been amenable to meta-analysis) in the GRADE table (Table S7). We explored the heterogeneity of included studies by ordering them according to populations, methodology, and outcomes reported. We prioritized the studies based on the assessed risk of bias, sample and effect size, and relevance to the research question. We included a description of synthesized findings for each outcome, including certainty of evidence with reference to p-values, odds ratios, and confidence intervals where available. We made conclusions based on the quality of included studies (risk of bias) and the certainty of evidence (Table S7).

## 3. Results

### 3.1. Characteristics of Included Studies

Of the 55 studies included in this review, there were 10 RCTs, four cohort studies, seven quasi-experimental (open, nonrandomized trial), 27 cross-sectional, five mixed-methods (pre-and post-intervention with qualitative component), and two qualitative studies. We used the World Bank income level classification to categorize the countries where the study was conducted [27]. Thirty-four studies were conducted in high-income countries (HIC): United Kingdom (*n* = 3), United States (*n* = 5), Australia (*n* = 3), Norway (*n* = 1), and Japan (*n* = 22). Twenty-one studies were conducted in low- and middle-income countries (LMIC): Indonesia (*n* = 3), Kenya (*n* = 3), Mongolia (*n* = 2), Pakistan (*n* = 2), and one study in Vietnam, South Africa, Brazil, Bangladesh, Palestine, Burundi, Uganda, Dominican Republic, Cambodia, Bosnia and Herzegovina, and one multi-country study. Table S4 shows the study settings, population descriptions, and interventions conducted.

We noted differences in the inclusion criteria of the study populations. Mothers’ enrollment period varied across the studies, from any time during pregnancy, childbirth, and the postpartum period. One study was conducted in 13 centers in eight countries and targeted both literate and illiterate mothers living in different geographical and cultural conditions and communities with low or easy access to health services [28]. In this review, most studies were conducted in health facilities, and a few were conducted in community settings.

We also noted differences in the type of interventions. Of 55 studies, 34 focused on the MCH handbook, and the remaining 21 focused on other home-based records, such as maternity case notes, road to health (RTH) booklet, personal child health record, among others. Some studies did not have a comparison group, while others were compared to standard care or non-users of home-based records. Hence, the results were reported considering home-based records as a single intervention. Overall, the studies were heterogeneous, and the sample sizes mainly varied (range: 5–5,940,204) (Table S4).

### 3.2. Risk of Bias in Included Studies

**Figure 2:**
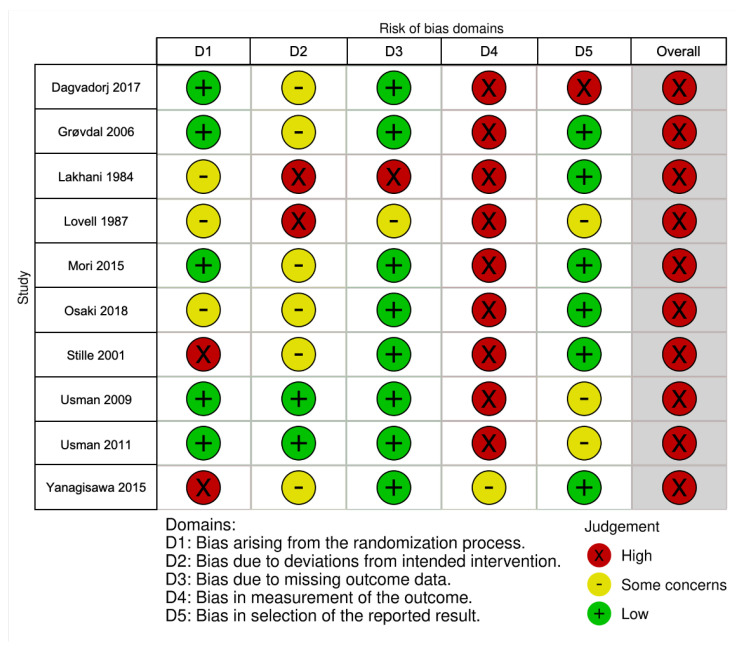
Risk of bias summary for randomized controlled trials based on authors’ judgments (low, some concerns, high) about each risk of bias item for each included study.

We noted differences in the risk of bias across the studies. Figure 2 shows the risk of bias assessment for the 10 RCTs included in this review [29]. Following the RoB 2 algorithm, all selected studies had a high overall risk of bias, mainly owing to bias from the randomization process, blinding/masking not described or not possible for the said intervention, and selective outcome reporting. For non-RCTs, the results of the risk of bias assessment are shown in Table S5. We found some methodological considerations and critical potential confounding variables neither measured nor adjusted statistically.

### 3.3. Roles of Home-Based Records on Newborn Health

Eight studies assessed the roles of home-based records on newborn health [6,7,28,30,31,32,33,34] (Table 1). Findings suggested a positive effect of home-based records on newborn care seeking (*n* = 2) and newborn care knowledge (*n* = 2). In Burundi, the proportion of mothers who sought postnatal care (PNC) from health personnel after delivery significantly increased from 35.9% to 64.2% after using the MCH handbook (*p* < 0.05, 95% CI: 59.2–69.3%) [30]. In South Africa, the proportion of HIV PCR birth tests registered with the RTH booklet identifier reached > 50% after only six months, suggesting that the RTH booklet can successfully be leveraged to provide infants with a unique patient identifier at birth [31]. In Indonesia, the mother class used the MCH handbook as the main reference material during the educational sessions [32]. Attending mothers showed improvement in knowledge regarding immediate breastfeeding (OR = 2.79, 95% CI: 1.48–5.25), giving colostrum (OR = 2.09, 95% CI: 1.09–4.02), exclusive breastfeeding duration (OR = 3.54, 95% CI: 2.04–6.15), use of antibiotic for newborn’s eyes (OR = 4.58, 95% CI: 3.05–6.88), hepatitis B immunization at birth (OR = 1.56; 95% CI: 1.06–2.28), thermal protection (OR = 3.99, 95% CI: 1.59–10.06), cord care (OR = 8.86, 95% CI: 5.69–13.8), and recognition of danger signs in newborns (OR = 3.29, 95% CI: 2.15–5.05) [32]. Indonesian mothers who used the MCH handbook were more likely to practice good newborn care than the standard care group (OR = 1.81, 95% CI: 1.24–2.66) [32]. Similarly, a multi-country study showed that mothers who used home-based maternal records became more involved in looking after their health and newborns [28].

However, findings suggest no significant effects of home-based records on immediate breastfeeding (*n* = 2), neonatal deaths (*n* = 2), or APGAR (i.e., appearance, pulse, grimace, activity, and respiration) score (*n* = 1). One RCT conducted in the UK showed no significant difference between mothers in the case note group and cooperation card group about breastfeeding after delivery (78.9% vs. 77.4%, respectively) and the incidence of neonatal deaths (both 2.0%, GRADE certainty of evidence: very low) [34]. The same results were obtained in one cluster RCT conducted in Mongolia [7]. A higher rate of immediate breastfeeding initiation was observed among mothers who received the MCH handbook (versus no handbook); however, there was no significant difference (RR = 1.07, 95% CI: 0.97–1.18, GRADE certainty of evidence: moderate) [7]. Moreover, there was no significant difference in either neonatal deaths (RR = 1.00, 95% CI: 0.99–1.02, GRADE certainty of evidence: very low) or APGAR scores (MD = 0.21, 95% CI: −0.21–0.63, GRADE certainty of evidence: moderate) using the MCH handbook compared with the control group [7].

### 3.4. Roles of Home-Based Records on Child Health

We found evidence on the positive effects of home-based records on childcare seeking (*n* = 6) and vaccination uptake and recording (*n* = 10) (Table 2).

Mothers who received home-based records were more likely to utilize healthcare services [6,51,52,53,54,55] and adhere to the recommended immunizations and childcare visit recommendations [42,43,44,45,46,47,48,49,50,56,57]. However, a mixed effect was obtained concerning age-appropriate immunization, such as a three-dose series of diphtheria, tetanus toxoids, and pertussis (DTP) [35,36,37,38]. Findings in randomized trials conducted in the UK and USA suggested no significant effects on the DTP3 completion rate among children using home-based records compared to no home-based records (pooled RR = 0.89, 95% CI: 0.64–1.24, GRADE certainty of evidence: very low) [35,36]. In contrast, in an RCT conducted in Pakistan, children who were using a redesigned immunization card showed significant improvement in the DTP3 completion rate compared to a standard expanded program on immunization (EPI) card (pooled RR = 1.46, 95% CI: 1.09–1.94, GRADE certainty of evidence: moderate) [37,38].

Six out of 16 studies showed a positive effect of home-based records on child healthcare knowledge. Mothers who used home-based records showed improvement in their knowledge of general health [51], immunization [56], exclusive breastfeeding [56,62,63], biliary atresia [65], and infant accident prevention [68]. Among less-educated Japanese women who are literate, the MCH handbook effectively provided adequate knowledge about exclusive breastfeeding [62]. Similarly, mothers who had read the MCH handbook were more careful of infant accidents than a group of mothers who had never read the handbook (*p* < 0.01) [68]. On the other hand, a few studies reported no effect/mixed results related to immunization [59,60,61], detection of biliary atresia [64,66], and sudden infant death syndrome [67]. Japanese mothers requested more information on the vaccination schedule, stool color card, and sudden infant death syndrome.

Most studies reported an improvement in child health practices (*n* = 8) and illness management (*n* = 3) among recipients of home-based records. A significant change in exclusive breastfeeding practice was seen among mothers who received the MCH handbook in Bangladesh (16.9% vs. 0.7%) and Vietnam (74.9% vs. 18.3%) [56,63]. However, one RCT conducted in Indonesia showed no effect of the MCH handbook on exclusive breastfeeding for six months (OR = 0.76, 95% CI: 0.51–1.14, GRADE certainty of evidence: low) [6]. Despite this opposite result, the authors reported that Indonesian mothers who received the MCH handbook tended to practice continued breastfeeding (OR = 2.31, 95% CI: 1.22–4.39), complementary feeding (OR = 4.35, 95% CI: 2.85–6.65), proper feeding order (OR = 2.70, 95% CI: 1.79–4.09), varied foods feeding, e.g., by providing fruits and/or fruits extract (OR = 2.18, 95% CI: 1.42–3.36), and self-feeding training (OR = 2.75, 95% CI: 1.74–4.36) [6] (GRADE certainty of evidence: moderate). In a similar study, mothers who received the MCH handbook tended to practice home care for cough (OR = 3.50, 95% CI: 1.44–8.52, GRADE certainty of evidence: low) and vitamin A use (OR = 2.00, 95% CI: 1.16–3.47, GRADE certainty of evidence: moderate) [6]. The study did not show a significant difference between the intervention and control arm for home care for diarrhea (83.3% of 24 cases, 92.6% of 27 cases) [6]. In contrast, growth monitoring seemed to concern mothers who received a home-based record. Mothers ignored developmental indicators that they did not understand, such as head circumference [72] and growth curves (e.g., weight-versus-age chart, BMI-versus-age chart) [73,75]. Further explanation may have supported mothers to address this issue. In Japan, reading and completing the MCH handbook were associated with maternal characteristics, with older mothers and mothers with little childcare experience completing the handbook more [74].

Two randomized studies showed evidence related to child mortality and morbidity [6,78]. In Indonesia, mothers who received the MCH handbook were less likely to have underweight children (OR = 0.33, 95% CI: 0.12–0.94, GRADE certainty of evidence: very low) and children with stunted growth (OR = 0.53, 95% CI: 0.30–0.92, GRADE certainty of evidence: low) [6]. However, no significant difference was observed between the intervention and control arm concerning wasting among children [6]. In Mongolia, MCH handbook users showed a reduction in the risk of children’s cognitive delay compared with the control group at a three-year follow-up (AOR = 0.32, 95% CI: 0.14–0.73, GRADE certainty of evidence: very low) [78].

Seven out of eight studies showed a positive effect of home-based records on the continuum of care [6,28,30,49,63,79,80]. Among all home-based records, the MCH handbook showed evidence of the continuum of maternal, newborn, and childcare. In Vietnam, the proportion of pregnant women who made ≥ 3 antenatal care (ANC) visits and practiced exclusive breastfeeding significantly increased between pre- and post-intervention *(p* < 0.001) [63]. In Burundi, the proportion of mothers who received birth notification at health facilities significantly increased from 4.6% to 61.0% (*p* < 0.05, 95% CI: 55.9–66.2%) [30]. In the Dominican Republic, the rate of mothers receiving antenatal and postpartum care at designated clinics or hospitals increased from 13% to 40% [49]. In Japan, mothers who had seen their own MCH handbook when they were young had a higher continuity awareness than those who had not [80]. The MCH handbook also strengthened the collaborative interactions among hospitals, local government units, and schools [81]. However, more effort is needed to translate the positive utilization of the MCH handbook into personal responsibility for health [81].

## 4. Discussion

Users of home-based records showed improvement in care seeking, knowledge, and practices of newborn/childcare. However, home-based records may not significantly affect the practice of immediate breastfeeding and prevention of perinatal mortality and morbidity. We also observed a mixed effect on age-appropriate immunization, such as DTP3 completion rate. Despite this inconsistency, home-based records provided essential data on the immunization status of the child. Furthermore, of all home-based records, the MCH handbook showed a positive effect on promoting the continuum of care and childhood illness management.

Home-based records (especially the MCH handbook) could improve mothers’ knowledge and practices of newborn/childcare. In this review, we found the positive result could be attributed to the following reasons: educational sessions, frequent use of records, and healthcare providers’ involvement. In Indonesia, mothers who attended the MCH handbook educational sessions were more likely to practice immediate breastfeeding, thermal protection, cord care, and recognize the danger signs in a newborn than those who did not [32]. This finding is new because Magwood et al. [3] reported no statistically significant effects on newborn health outcomes. The difference in results is due to not including quasi-experimental studies in their selection criteria. Moreover, Indonesian mothers read and brought the MCH handbook to multiple healthcare facilities on different occasions [6]. Multiple healthcare providers recorded health information in the same handbook, enabling more frequent monitoring of the child’s health [6]. This frequent use and consultation might have facilitated the sharing of information between healthcare providers and mothers. Consequently, mothers become more motivated and aware of their children’s health and needs and are more likely to translate their knowledge into practice. They tended to practice proper feeding (e.g., exclusive breastfeeding, complementary feeding, to name a few), vitamin A use, and home care for cough [6]. There were fewer children with stunted growth or underweight among users of the handbook compared to non-users [6]. In Mongolia, the risk of cognitive delay is lower among users of the handbook, possibly because mothers were more likely to be concerned about developmental milestones and interacting with their children more [78]. These review findings suggest that the MCH handbook may reduce newborn/child morbidity and facilitate growth and development. This shows the potential of the handbook to be combined with the integrated management of newborn and childhood illness (IMNCI). The handbook may complement the IMNCI global strategy of promoting a holistic, child-centered approach to childhood illness [82].

Users of home-based records showed poor knowledge and practice related to immunization, biliary atresia, sudden infant death syndrome, and growth monitoring. These findings are new and warrant further attention. In Japan, the low measles vaccine coverage rate may be due to mothers’ misunderstanding and lack of information, including their fear of side effects [60,61]. In addition, Japanese mothers cannot understand how the stool color card works to detect biliary atresia [64,66] and have never heard of sudden infant death syndrome [67]. Although they showed a high interest in the disease, further information and explanation might help properly use the stool color card. In Australia and Brazil, growth monitoring was a concern among mothers. Typically, mothers ignored developmental indicators (e.g., head circumference, weight-versus-age chart, BMI-versus-age chart) that they did not understand [72,73]. The findings in this review highlighted the importance of providing adequate written information in the home-based records and an explanation from the healthcare providers to support mothers’ understanding.

While home-based records improved newborn/childcare seeking, as shown by the utilization of healthcare services, we found inconsistencies in receiving age-appropriate immunization. Two RCTs in the UK and USA suggested no significant effects on the DTP3 completion rate among children using home-based records compared to no home-based records [35,36]. However, in Pakistan, two RCTs revealed a significant improvement in the DTP3 completion rate with newly designed immunization cards and educational interventions versus the standard EPI card [37,38]. Magwood et al. [3] reported the same findings in their systematic review. The review findings offer moderate evidence that an improved home-based record and center-based education (designed for the low literacy population) effectively educated parents in LMIC concerning immunization uptake, as reported in Pakistan. In general, home-based records provided essential data on the child’s immunization status, regardless of the income status [42,43,44,45,46,47,48,49,50]. A higher proportion of children using a home-based record had up-to-date immunization status compared to non-users, which implies the potential of the records to encourage utilization of healthcare services and keep track of children’s immunization history in both LMIC and HIC. This review finding contrasts with another review [83] that reported a positive relationship between the use of home-based records and child immunization uptake in LMIC, but not in HIC. The difference can be accounted for by the available evidence presented in the review.

Home-based records showed no significant effect on the practice of immediate breastfeeding and prevention of perinatal mortality and morbidity. While we observed in this review improvement in “knowledge” of immediate breastfeeding among MCH handbook users in Indonesia [32] and Cambodia [33], we did not see a significant difference in the “practice” of immediate breastfeeding in the UK (maternity case notes vs. standard card) [34] and Mongolia (MCH handbook vs. no handbook) [7]. We need to interpret this result carefully because statistically speaking, there was no effect, but the proportion/rate of mothers who practiced immediate breastfeeding was higher in the intervention group. Other factors might have influenced the results. For instance, in the UK trial, mothers in the control group (standard card) had access to their case notes during their healthcare visits and may have benefitted from access to this information [34]. In the Mongolia trial, there was no masking, and recall bias likely exists in the analysis, because data collection was conducted one month after birth [7]. Finally, home-based records showed no effect on stillbirth or neonatal death [7,34] and no difference in APGAR scores [7]. Both trials reported no more than two still births/neonatal deaths between the intervention and control groups, suggesting a negligible effect size.

Of all home-based records, the MCH handbook provides evidence of facilitating the continuum of care. The handbook promotes the uptake of multiple services from pregnancy to early child-rearing stages. Magwood et al. [3] reported the same finding in Indonesia, where mothers reported additional benefits of having skilled birth attendants during delivery and observed proper feeding practices [6]. We found more evidence on the role of the handbook in the continuum of care. In the Dominican Republic, the handbook was well accepted by pregnant women for its simplicity, friendliness, durability, and mobility, and the rate of receiving ANC and PNC at designated clinics or hospitals increased [49]. The handbook may also ensure the well-being of the next generation of parents. Japanese mothers who had seen their handbook when they were young were more likely to share the handbook as a gift for their children during marriage or pregnancy [80]. The handbook has also strengthened the collaboration among hospitals, local governments, and schools in Japan [81]. These findings are new and require further investigation. Home-based records, particularly the MCH handbook, may enhance communication and ensure the utilization of healthcare services from pregnancy to childhood (continuum of care) and pass on healthy behaviors to the next generation. These findings further show the relative advantage of using an integrated home-based record (e.g., MCH handbook) over stand-alone records (e.g., growth charts, vaccination cards).

This systematic review has several limitations. First, findings from observational studies should always be interpreted with caution as their potential biases are greater than RCTs. Our initial objective was to go beyond conducting only an RCT-based review; thus, we included observational studies to show the effects of home-based records on newborn and child health. The Cochrane handbook also suggested the strengths of including observational studies when the review question cannot be answered by RCTs completely [84]. Second, we included a broad range of outcomes, and only a few studies were available for each outcome category. The number of available studies was insufficient to conduct subgroup analyses to compare HIC and LMIC. Third, there was marked heterogeneity in the study populations, types of interventions, comparator groups, and outcome measurements—all of which may modify the effect of the interventions. The 10 RCTs included in this review had an overall high risk of bias due to the lack of blinding. Owing to such heterogeneity and/or bias, we conducted a narrative synthesis to explain the effect of home-based records and presented the GRADE table to show the quality assessment and certainty of evidence. Fourth, the available evidence was insufficient to conduct a network meta-analysis to assess the relative advantages of the different home-based records, making the comparisons difficult. Thus, we summarized the effect of the intervention based on the type of home-based records used in the study. Despite these limitations, this systematic review has its strengths in its comprehensive nature in the search strategy and data analysis and examining original research articles published in the Japanese language. We included a relatively large number of studies (*n* = 55) of all study designs compared to the previous review (*n* = 9) that included only RCTs and studies written in English. Therefore, readers can see the bigger picture to understand better the roles of home-based records on newborn and child health.

## 5. Implications for Future Research

The review found a limited availability of RCTs regarding the roles of home-based records on newborn and child health. While we cannot undermine the value of the findings obtained from observational studies, the certainty of the evidence is between very low and low, which limits policy recommendations. Nevertheless, observational studies highlighted the immense contribution of home-based records to improve newborn and child health outcomes. Further research is needed to explore the complex contribution of home-based records in improving the quality of newborn/childcare. Moving forward, the findings in this review emphasized the role of home-based records as excellent health promotion tools. It calls for adequate written health information to resolve mothers’ fear and/or lack of understanding about particular vaccines, growth charts, and disease conditions, such as biliary atresia and sudden infant death syndrome. Improving the design features and providing educational sessions were effective interventions to improve the uptake of health services, especially in LMIC. Healthcare providers are encouraged to use the records during consultations to support mothers in their pregnancy and early child-rearing stages. The frequent use of the records may also have facilitated the uptake of healthcare services. As we can use home-based records in the management of sick children, it is worthy of making it a component of the IMNCI in addressing newborn/childhood illnesses. Further research is needed to measure the effectiveness of the combined approach of home-based records and IMNCI in improving newborn/childcare.

## 6. Conclusions

The MCH handbook and other home-based records may be used as practical and valuable tools in improving newborn and child health outcomes, regardless of socio-economic conditions. Users of home-based records showed improvement in care seeking, knowledge, and practices of newborn/childcare. Home-based records are excellent health promotion tools and may reduce child morbidity and improve the continuum of care. We did not find any harm associated with the use of home-based records. Studies included in this review did not report the adverse effects of home-based records. Nevertheless, we recommend standardizing the records and integrating them into the national guideline of pregnancy registration and antenatal care. Involving local governments, hospitals, and schools can help ensure the successful uptake of the records and utilization of MNCH services. Home-based records are indispensable tools to ensure that newborns and children are not left behind in the era of SDGs.

## Figures and Tables

**Table 1:** Roles of the MCH handbook and other home-based records on newborn health.

Outcome	Intervention	No Effect, (*n*)	Mixed Effect, (*n*)	Positive Effect, *n* (%)	Total Times Outcome Reported, (*n*)
**Newborn care seeking**					
Care seeking for newborn complications [6]	MCH handbook	1	0	0 (0)	1
Care seeking after delivery [30]	MCH handbook	0	0	1 (100)	1
Use of laboratory services [31]	RTH booklet	0	0	1 (100)	1
Total (newborn care seeking)		1	0	2 (67)	3
**Newborn care knowledge**					
Immediate breastfeeding [32,33]	MCH handbook	0	0	2 (100)	2
Giving colostrum [32]	MCH handbook	0	0	1 (100)	1
Exclusive breastfeeding Duration [32]	MCH handbook	0	0	1 (100)	1
Use of antibiotic for eyes [32]	MCH handbook	0	0	1 (100)	1
Hepatitis B immunization at birth [32]	MCH handbook	0	0	1 (100)	1
Thermal protection [32]	MCH handbook	0	0	1 (100)	1
Cord care [32]	MCH handbook	0	0	1 (100)	1
Recognize the danger signs in newborns [32]	MCH handbook	0	0	1 (100)	1
Total (newborn care knowledge)		0	0	9 (100)	9
**Newborn care practices**					
Immediate breastfeeding [7,34]	MCH handbook; maternity case notes	2	0	0 (0)	2
Good newborn care and self-care [28,32]	MCH handbook; home-based maternal record	0	0	2 (100)	2
Total (newborn care practices)		2	0	2 (50)	4
**Perinatal mortality and morbidity**					
Neonatal deaths [7,34]	MCH handbook; maternity case notes	2	0	0 (0)	2
APGAR score [7]	MCH handbook	1	0	0 (0)	1
Total (perinatal mortality and morbidity)		3	0	0 (0)	3

MCH—maternal and child health, APGAR—appearance, pulse, grimace, activity, and respiration, RTH—road to health.

**Table 2:** Roles of the MCH handbook and other home-based records on child health.

Outcome	Intervention	No Effect, (*n*)	Mixed Effect, (*n*)	Positive Effect, *n* (%)	Total Times Outcome Reported, (*n*)
**Vaccination use/uptake**					
DTP3 completion [35,36,37,38]	Home-based health record booklet; educational immunization cards; redesigned immunization card	2	0	2 (50)	4
Rotavirus vaccination [39]	MCH handbook	0	1	0 (0)	1
BCG and polio vaccination [40]	MCH handbook	0	1	0 (0)	1
Mumps, measles, chickenpox [41]	MCH handbook	0	1	0 (0)	1
Total (vaccination use/uptake)		2	3	2 (29)	7
**Vaccination history/records** [42,43,44,45,46,47,48,49,50]	MCH handbook; personal health record; personal child health record and advice booklet; individually tailored calendar; child health card; patient-held vaccination records; personalized calendar	0	1	8 (89)	9
**Childcare seeking**					
Care seeking for child Illnesses [6,51,52,53]	MCH handbook; personal health record; parent-held child health record	2	0	2 (50)	4
Use of healthcare and laboratory Services [53,54,55]	MCH handbook; parent-held child health record	1	0	2 (67)	3
Adherence to recommended Immunizations [56,57]	MCH handbook; personal health record	0	0	2 (100)	2
Adherence to childcare visit Recommendations [57]	Personal health record	0	0	1 (100)	1
Total (childcare seeking)		3	0	7 (70)	10
**Child healthcare knowledge**					
General health [51,53,58]	MCH handbook; personal child health record; parent-held child health record	2	0	1 (33)	3
Immunization [32,56,59,60,61]	MCH handbook	3	1	1 (20)	5
Exclusive breastfeeding [56,62,63]	MCH handbook	0	0	3 (100)	3
Detection of biliary atresia [64,65,66]	MCH handbook stool card	0	2	1 (33)	3
Sudden infant death syndrome [67]	MCH handbook	0	1	0 (0)	1
Accident prevention of infant [68]	MCH handbook	0	0	1 (100)	1
Total (child healthcare knowledge)		5	4	7 (44)	16
					
**Child healthcare practices**					
Exclusive breastfeeding [6,56,63]	MCH handbook	1	0	2 (67)	3
Continued breastfeeding [6]	MCH handbook	0	0	1 (100)	1
Complementary feeding [6]	MCH handbook	0	0	1 (100)	1
Proper feeding order [6]	MCH handbook	0	0	1 (100)	1
Varied foods feeding [6]	MCH handbook	0	0	1 (100)	1
Complementary feeding [6]	MCH handbook	0	0	1 (100)	1
Proper feeding order [6]	MCH handbook	0	0	1 (100)	1
Varied foods feeding [6]	MCH handbook	0	0	1 (100)	1
Self-feeding training [6]	MCH handbook	0	0	1 (100)	1
Recording immunizations [35,45,69,70,71]	MCH handbook; home-based health record booklet; child health record; home-based record	1	2	2 (40)	5
Growth monitoring [45,70,72,73,74,75,76,77]	MCH handbook; child health card; child personal health record; child health handbook; health and living log	3	2	4 (44)	9
Total (child healthcare practices)		5	4	13 (59)	22
**Infant and child illness management**					
Home care for cough [6]	MCH handbook	0	0	1 (100)	1
Home care for diarrhea [6,49]	MCH handbook	1	0	1 (50)	2
Vitamin A use [6,56]	MCH handbook	0	0	2 (100)	2
Total (infant and child illness management)		1	0	4 (80)	5
**Child mortality and morbidity**					
Underweight children [6]	MCH handbook	0	0	1 (100)	1
Stunted growth [6]	MCH handbook	0	0	1 (100)	1
Wasting [6]	MCH handbook	1	0	0 (0)	1
Risk of cognitive delay [78]	MCH handbook	0	0	1 (100)	1
Total (child mortality and morbidity)		1	0	3 (75)	4
**Continuum of care** [6,28,30,49,63,79,80,81]	MCH handbook; home-based maternal record	0	1	7 (88)	8

MCH—maternal and child health, DTP3—third dose of diphtheria, tetanus toxoids, and pertussis, BCG—Bacillus Calmette–Guérin.

## Data Availability

All relevant data are within the manuscript and its Supporting Information files.

## Supplementary Materials

The following are available online at https://www.mdpi.com/article/10.3390/ijerph18147463/s1, Text S1: PROSPERO registration, Text S2: Search strategy for English and Japanese articles, Table S1: Table of excluded studies with reasons, Table S2: List of English articles, Table S3: List of Japanese articles, Table S4: PICO table, Table S5: Risk of bias for observational studies, Table S6: SWiM checklist, Table S7: GRADE table.
