# Supplementary material for: Roles of the Maternal and Child Health Handbook and Other Home-Based Records on Newborn and Child Health: A Systematic Review
Source: Int J Environ Res Public Health. 2021 Jul 13;18(14):7463. doi: 10.3390/ijerph18147463 (PMC8306696; doi:10.3390/ijerph18147463)
Supplement: Supplementary file 1 [file ijerph-18-07463-s001.zip › Table S1. Excluded studies.pdf]

Table S1. Table of excluded studies with reasons

No.	Study ID	Reasons for exclusion
1	Abughali 2014	Electronic medical records
2	Adams 2013	Electronic medical records
3	Aiga 2016	Not related to newborn/child health outcomes
4	Aiga 2018	Not related to newborn/child health outcomes
5	Aihara 2006	Not related to newborn/child health outcomes
6	Akashi 2018	Not related to home-based records nor the MCH handbook
7	Akhund 2011	Not related to newborn/child health outcomes
8	Akiba 2016	Not related to newborn/child health outcomes
9	Albers 1997	Electronic medical records
10	Angier 2014	Electronic medical records
11	Balakrishnan 2016	Mobile health intervention
12	Baqui 2019	Not related to home-based records nor the MCH handbook
13	Bartsch 2018	Electronic medical records
14	Belemsaga 2018	Not related to home-based records nor the MCH handbook
15	Bellows 2013	Not related to home-based records nor the MCH handbook
16	Bilenko 2017	Not related to home-based records nor the MCH handbook
17	Boothroyd 2011	Not related to home-based records nor the MCH handbook
18	Bose 2015	Electronic medical records
19	Braeye 2019	Electronic medical records
20	Bremberg 2000	Not related to home-based records nor the MCH handbook
21	Brodgribb 2016	Not related to home-based records nor the MCH handbook
22	Bryanton 2013	Not related to home-based records nor the MCH handbook
23	Bundy 2013	Electronic medical records
24	Carsley 2018	Electronic medical records
25	Chung 2018	Electronic medical records
26	Clancy 2013	Electronic medical records
27	Clendon 2010	Not related to newborn/child health outcomes
28	Coleman 2017	Mobile health intervention
29	de Hoon 2017	Electronic medical records
30	DeVoe 2018	Electronic medical records
31	Du Plessis 2017	Not related to newborn/child health outcomes
32	Elbourne 1987	Not related to newborn/child health outcomes
33	Ferreccio 2008	Not related to home-based records nor the MCH handbook
34	Fiks 2006	Electronic medical records
35	Fiks 2012	Electronic medical records
36	Fiks 2015	Electronic medical records
37	Franchetti 2014	Not related to home-based records nor the MCH handbook
38	Froen 2016	Electronic medical records
39	Fukuda 2019	Not related to home-based records nor the MCH handbook
40	Fukushima 2016	Not related to home-based records nor the MCH handbook
41	Guyer 2000	Not related to home-based records nor the MCH handbook
42	Hagelin 1998	Not related to home-based records nor the MCH handbook
43	Haider 2017	Not related to home-based records nor the MCH handbook
44	Hampshire 2004	Not related to newborn/child health outcomes
45	Hasegawa 2015	Not related to home-based records nor the MCH handbook

46	Hawley 2014	Electronic medical records
47	Helle 2019	Electronic medical records
48	Hidechika 2018	Not related to home-based records nor the MCH handbook
49	Higashiyama 2013	Not related to newborn/child health outcomes
50	Hiura 2002	Not related to home-based records nor the MCH handbook
51	Irwanto 2019	Not related to newborn/child health outcomes
52	Julius 2013	Not related to newborn/child health outcomes
53	Kelaher 2009	Not related to home-based records nor the MCH handbook
54	Kelle 2015	Electronic medical records
55	Khresheh 2008	Electronic medical records
56	Kitayama 2014	Electronic medical records
57	Kubota 2000	Not related to home-based records nor the MCH handbook
58	Kusumayati 2007	Not related to newborn/child health outcomes
59	Lain 2009	Not related to home-based records nor the MCH handbook
60	Lansdown 1996	Not related to home-based records nor the MCH handbook
61	Leppert 1993	Not related to home-based records nor the MCH handbook
62	Liabsuetrakul 2017	Electronic medical records
63	Liberato 2016	Electronic medical records
64	Little 2013	Mobile health intervention
65	Luman 2009	Not related to newborn/child health outcomes
66	Lund 2016	Mobile health intervention
67	Lupton 2017	Mobile health intervention
68	Lwembe 2016	Not related to home-based records nor the MCH handbook
69	Mahanta 2016	Not related to home-based records nor the MCH handbook
70	Markellis 1973	Not related to home-based records nor the MCH handbook
71	Matsumoto 1996	Not related to newborn/child health outcomes
72	Matsushita 2011	Not related to newborn/child health outcomes
73	Mawarni 2017	Electronic medical records
74	McKinn 2017	Not related to newborn/child health outcomes
75	Mengoni 2014	Not related to home-based records nor the MCH handbook
76	Minewaki 2019	Not related to newborn/child health outcomes
77	Miyake 2018	Not related to newborn/child health outcomes
78	Nishi 1990	Not related to home-based records nor the MCH handbook
79	Naito 2019	Not related to newborn/child health outcomes
80	Odai 2014	Not related to home-based records nor the MCH handbook
81	O’Flaherty 1987	Not related to newborn/child health outcomes
82	Okawa 2019	Not related to home-based records nor the MCH handbook
83	Okereke 2015	Not related to home-based records nor the MCH handbook
84	Ooki 2005	Not related to home-based records nor the MCH handbook
85	Osaka 1995	Not related to home-based records nor the MCH handbook
86	Panagiotou 1998	Electronic medical records
87	Pies 2012	Not related to home-based records nor the MCH handbook
88	Polnay 1989	Not related to newborn/child health outcomes
89	Popovich 2008	Not related to home-based records nor the MCH handbook
90	Pratinidhi 2015	Not related to home-based records nor the MCH handbook
91	Rahman 2016	Not related to home-based records nor the MCH handbook
92	Ramraj 2018	Not related to newborn/child health outcomes
93	Reddaiah 1985	Not related to newborn/child health outcomes

94	Reich 2010	Not related to home-based records nor the MCH handbook
95	Riverin 2015	Electronic medical records
96	Rourke 2009	Electronic medical records
97	Rourke 2010	Electronic medical records
98	Rourke 2013	Electronic medical records
99	Sachs 2011	Not related to newborn/child health outcomes
100	Sadiq Sheikh 2014	Not related to newborn/child health outcomes
101	Saeedzai 2019	Not related to newborn/child health outcomes
102	Seto 2006	Not related to newborn/child health outcomes
103	Shimada 2017	Not related to home-based records nor the MCH handbook
104	Spencer 2000	Not related to home-based records nor the MCH handbook
105	Stanton 2013	Not related to home-based records nor the MCH handbook
106	Sugi 1985	Not related to newborn/child health outcomes
107	Takahashi 2007	Not related to newborn/child health outcomes
108	Takeda 2002	Not related to newborn/child health outcomes
109	Takehara 2016	Not related to home-based records nor the MCH handbook
110	Takeuchi 2014	Not related to home-based records nor the MCH handbook
111	Talbott 2015	Not related to home-based records nor the MCH handbook
112	Tamburlini 2011	Not related to home-based records nor the MCH handbook
113	Tamburlini 2013	Not related to home-based records nor the MCH handbook
114	Tanabe 2011	Not related to newborn/child health outcomes
115	Thomas 2011	Not related to home-based records nor the MCH handbook
116	Tobe 2018	Mobile health intervention
117	Tunçalp 2013	Not related to home-based records nor the MCH handbook
118	Umeda 2015	Not related to newborn/child health outcomes
119	Uneke 2017	Not related to home-based records nor the MCH handbook
120	Uneke 2018	Not related to home-based records nor the MCH handbook
121	Vanosdoll 2019	Mobile health intervention
122	Vincelet 2003	Not related to newborn/child health outcomes
123	Vinceten 2012	Not related to home-based records nor the MCH handbook
124	Walton 2006	Not related to newborn/child health outcomes
125	Walton 2007	Not related to newborn/child health outcomes
126	Wilkinson 2010	Not related to home-based records nor the MCH handbook
127	Wilcox 2019	Mobile health intervention
128	Wilson 2014	Mobile health intervention
129	Wright 2005	Not related to newborn/child health outcomes
130	Young 1990	Not related to newborn/child health outcomes
