# Supplementary material for: Roles of the Maternal and Child Health Handbook and Other Home-Based Records on Newborn and Child Health: A Systematic Review
Source: Int J Environ Res Public Health. 2021 Jul 13;18(14):7463. doi: 10.3390/ijerph18147463 (PMC8306696; doi:10.3390/ijerph18147463)
Supplement: Supplementary file 1 [file ijerph-18-07463-s001.zip › Table S2. English articles.pdf]

Table S2. English articles showing the effects of the MCH handbook and other home-based records on newborn and child health

Outcomes	Reference<br>Study design	Intervention	Effect of intervention	Comments
**Newborn care-seeking**				
Care-seeking for newborn complications	Osaki, 2018<br>Cluster RCT	MCH handbook	No impact	Respondents in the intervention arm were more likely to seek care for newborn complications (3.3 vs. 10.3%), although difference was non-significant.
Care-seeking after delivery	Kaneko, 2017<br>Quasi-experimental	MCH Handbook	Positive	The proportion of mothers who received guidance on PNC from health personnel after delivery significantly increased (*p*-value <0.05) from 35.9% to 64.2% (95% CI: 59.2-69.3%)
Use of laboratory services	Haeri Mazanderani, 2018<br>Retrospective cohort	RTH booklet	Positive	The proportion of HIV PCR birth tests registered with the RTH booklet identifier reached >50% after only six months, suggesting RTH booklet can successfully be leveraged to provide infants with a unique patient identifier at birth. The RTH booklet identifiers can readily be incorporated in the laboratory information system.
**Newborn care knowledge**				
Immediate breastfeeding	Nasir, 2017<br>Quasi-experimental	MCH handbook	Positive	Mothers in the intervention group knew that breastfeeding should be initiated within 1 h of birth (OR=2.79, 95% CI: 1.48-5.25).
	Yanagisawa, 2015<br>RCT	MCH handbook	Positive	Mothers who received the MCH handbook showed improvement in knowledge of early breastfeeding (effect= 6.2)
Giving colostrum	Nasir, 2017<br>Quasi-experimental	MCH handbook	Positive	Mothers in the intervention group showed improvement in knowledge of giving colostrum (OR=2.09, 95% CI: 1.09-4.02).
Exclusive breastfeeding duration	Nasir, 2017<br>Quasi-experimental	MCH handbook	Positive	Mothers in the intervention group understood the recommended duration of exclusive breastfeeding (OR=3.54, 95% CI: 2.04-6.15).
Use of antibiotic for eyes	Nasir, 2017<br>Quasi-experimental	MCH handbook	Positive	Mothers in the intervention group understood the use of antibiotic for newborn's eyes (OR=4.58, 95% CI: 3.05-6.88).
Hepatitis B immunization at birth	Nasir, 2017<br>Quasi-experimental	MCH handbook	Positive	Mothers in the intervention group showed significant increase in knowledge of Hepatitis B immunization at birth (OR=1.56; 95% CI: 1.06-2.28).
Thermal protection	Nasir, 2017<br>Quasi-experimental	MCH handbook	Positive	Mothers in the intervention group knew about thermal protection for newborns (OR=3.99, 95% CI: 1.59-10.06).
Cord care	Nasir, 2017<br>Quasi-experimental	MCH handbook	Positive	Mothers in the intervention group understood cord care for newborns (OR=8.86, 95% CI: 5.69-13.8).
Danger signs of newborn	Nasir, 2017<br>Quasi-experimental	MCH handbook	Positive	Mothers in the intervention group showed significant improvement in recognition of danger signs in newborns (OR=3.29, 95% CI: 2.15-5.05).
**Newborn care practices**				
Immediate breastfeeding	Lovell, 1987<br>RCT	Maternity case notes	No impact	No significant difference was observed between mothers in the case note group and cooperation card group with regard to immediate breastfeeding (78.9% vs. 77.4%).
	Mori, 2015<br>Cluster RCT	MCH handbook	No impact	A higher rate of early breastfeeding initiation was observed among mothers who received the MCH handbook (versus no handbook), but there was no significant difference (RR=1.07, 95% CI: 0.97-1.18).
Good newborn care and self-care	Shah, 1993<br>Quasi-experimental	Home-based maternal record	Positive	Mothers in the intervention group became more involved in looking after their own health and that of their babies.
	Nasir, 2017<br>Quasi-experimental	MCH handbook	Positive	Mothers in the intervention group were more likely to practice good newborn care compared with the control group (OR=1.81, 95% CI: 1.24-2.66).

Table S2. (continued)

Outcomes	Reference Study design	Intervention	Effect of intervention	Comments
**Perinatal mortality and morbidity**				
Neonatal deaths	Lovell, 1987 RCT	Maternity case notes	No impact	No significant difference was observed between mothers in the case note group and cooperation card group with regard to incidence of neonatal deaths (2.0% vs. 2.0%).
	Mori, 2015 Cluster RCT	MCH handbook	No impact	No significant difference in neonatal deaths with the use of MCH handbook compared with the control group (RR=1.00, 95% CI: 0.99-1.02).
APGAR score	Mori, 2015 Cluster RCT	MCH handbook	No impact	No significant difference in APGAR scores between users of MCH handbook and control group (MD= 0.21, 95% CI: -0.21-0.63).
**Vaccination use/uptake**				
DTP3 completion	Lakhani, 1984 RCT	Home-based health record booklet	No impact	No significant difference in DTP3 completion between users and non-users of home-based health record booklet.
	Stille, 2001 RCT	Educational immunization cards	No impact	No significant difference in DTP3 completion between users and non-users of educational immunization cards.
	Usman, 2009 RCT	Redesigned immunization card	Positive	DTP3 completion improved by 25% in redesigned card group compared with the standard care (ARR=1.25, 95% CI: 1.11-1.40). Moreover, DTP3 completion improved by 31% in redesigned card and education group compared with the standard care (ARR=1.31, 95% CI: 1.18-1.46).
	Usman, 2011 RCT	Redesigned immunization card	Positive	Higher proportion of children completed DTP3 in redesigned card group (61%) compared with the standard care (crude RR=1.70, 95% CI: 1.5, 2.0). Moreover, the same pattern was observed (66%) in redesigned card and education group compared with the standard care (crude RR=1.7, 95% CI: 1.40-2.00).
**Vaccination history/ records**	Jeffs, 1994 Quasi-experimental	Personal health record	Positive	Of the personal health record examined, 91% (95% CI: 0.80-1.00) had at least one immunization recorded, and 68% (95% CI: 0.60-0.76) had all recommended immunizations documented by the age of four years.
	McMaster, 1996 Cross-sectional	Personal child health record and advice booklet	Mixed	Provided essential data on immunization status of displaced children. However, several children whose mothers said that their child had received BCG, even some with a record of the immunization, had no scar.
	Kreuter, 2004 Quasi-experimental	Individually tailored calendar	Positive	A higher proportion of babies in the intervention group had up-to- date immunization status than the control group at the end of a nine-month enrollment period (82% vs. 65%, *p*-value <0.001) and at age 24 months (66% vs. 47%, *p*-value <0.001).
	Mukanga, 2006 Cross-sectional	Child health card	Positive	Children whose cards were seen were 10 times as likely to be up to date with the immunization schedule than those who did not have a card (OR=9.55, 95% CI: 3.19-29.45).
	McElligott, 2010 Cross-sectional	Patient-held vaccination records	Positive	Having a vaccination record was associated with a 62% increase in the odds of being up to date in immunization status, compared with not having a vaccination record (OR=1.62, 95% CI: 1.49-1.77).
	Abbott, 2013 Mixed methods	Personalized calendar	Positive	The average delay in those who received the calendar at their previous visit was 0.6 months (95% CI: -0.8-2.6) after the due date, compared to 3.3 months (95% CI: -0.6-7.5) in those who did not.

Table S2. (continued)

Outcomes	Reference<br>Study design	Intervention	Effect of intervention	Comments
**Child care-seeking**				
Care-seeking for child illnesses	Kawakatsu, 2015<br>Cross-sectional	MCH handbook	Positive	Impact of 9.4 and 12.6 percentage points for proper health-seeking behavior for fever and diarrhea, respectively, were statistically significant among users of the MCH handbook.
	Zhou, 2015<br>Retrospective cohort	Personal health record	Positive	PHR-registered children, compared with propensity score-matched non-registered children, had 21% (95% CI: 14-28; *p*-value <0.001) more outpatient clinic visits and 26% (95% CI: 16-37; *p*-value <0.001) more telephone encounters.
	Osaki, 2018<br>Cluster RCT	MCH handbook	No impact	Among the reported child illnesses (75.8 vs. 71.2%), care-seeking from health personnel was similarly observed in both intervention and control arms.
	Grøvdal, 2006<br>RCT	Parent-held child health record	No impact	For children with chronic diseases, 17% more parents in the control group visited the child health center than a group receiving the parent-held child health record. No test for significance was reported.
Use of health care and laboratory services	Grøvdal, 2006<br>RCT	Parent-held child health record	No impact	No significant difference in the utilization of health care services between parent-held child health record and control groups (non-routine care, *p*-value =0.58; specialist or hospital care, *p*-value =0.84.).
	Mudany, 2015<br>Cross-sectional	MCH handbook	Positive	HIV DNA testing in infants rose from 27,000 in 2007 to 55,000 in 2010 to 60,000 in 2012, which represents approximately 60% coverage of estimated HIV-exposed infants.
Adherence to the recommended immunizations	Bhuiyan, 2006<br>Mixed methods	MCH handbook	Positive	Mothers in the MCH handbook group showed significant improvement in child vaccination as compared with the control group (8.3% vs. 1.5%; *p*-value <0.05).
	Tom, 2014<br>Retrospective cohort	Personal health record	Positive	Children whose parents used one or more PHR feature (vs. none) had higher odds of adhering to the recommended immunizations only at KP Northwest (OR=1.2, 95% CI: 1.0-1.3).
Adherence to childcare visit recommendations	Tom, 2014<br>Retrospective cohort	Personal health record	Positive	PHR use was associated with better adherence to well-child care visit recommendations for both KP Hawaii (OR=1.9, 95% CI: 1.3-2.9) and KP Northwest (OR=2.5, 95% CI: 2.1-2.9).
**Child health care knowledge**				
General health	Moore, 2000<br>Quasi-experimental	Personal child health record	No impact	No evidence that the record improved the parent's perception of their child's general health care. No data were presented.
	Grøvdal, 2006<br>RCT	Parent-held child health record	No impact	No significant difference in the parents' knowledge about child health matters (*p*-value = 0.84) and illness (*p*-value = 0.22) between parent-held child health record and control groups.
	Kawakatsu, 2015<br>Cross-sectional	MCH handbook	Positive	Impact of 5.9 percentage points for higher health knowledge was statistically significant among users of the MCH handbook.
Immunization	Bhuiyan, 2006<br>Mixed methods	MCH handbook	Positive	Significant improvement in knowledge of immunization was seen among mothers who received the MCH handbook compared with the control group (32.4% vs. 5.7%; *p*-value <0.05).
	Nasir, 2017<br>Quasi-experimental	MCH handbook	No impact	Mothers in the intervention group did not show improvement in the knowledge of importance of immunization (OR=0.60, 95% CI: 0.14-2.53).

Table S2. (continued)

Outcomes	Reference<br>Study design	Intervention	Effect of intervention	Comments
Exclusive breastfeeding	Bhuiyan, 2006<br>Mixed methods	MCH handbook	Positive	Significant improvement in knowledge of breastfeeding was seen among mothers who received the MCH handbook compared with the control group (28.7% vs. 4.6%; *p*-value <0.05).
	Hagiwara, 2013<br>Quasi-experimental	MCH handbook	Positive	Among less educated women who are literate, the MCH handbook is effective in providing adequate knowledge about exclusive breastfeeding (*p*-value <0.05).
	Aiga, 2016<br>Mixed methods	MCH handbook	Positive	The proportion of pregnant women and mothers who correctly know exclusive breastfeeding significantly increased from 66.1% in pre-intervention to 86.7% in post-intervention (*p*-value <0.001).
**Child health care practices**				
Exclusive breastfeeding	Bhuiyan, 2006<br>Mixed methods	MCH handbook	Positive	Significant change in the practice of breastfeeding was seen among mothers who received the MCH handbook compared with the control group (16.9% vs. 0.7%; *p*-value <0.05).
	Aiga, 2016<br>Mixed methods	MCH handbook	Positive	The proportion of mothers who exclusively breastfed their children until six months of age significantly increased from 18.3% in pre-intervention to 74.9% in post-intervention (*p*-value <0.001).
	Osaki, 2018<br>Cluster RCT	MCH handbook	No impact	No significant difference in exclusive breastfeeding for 6 months between the intervention and control arms (OR=0.76, 95% CI: 0.51-1.14).
Continued breastfeeding	Osaki, 2018<br>Cluster RCT	MCH handbook	Positive	Respondents in the intervention arm were more likely to practice continued breastfeeding (OR=2.31, 95% CI: 1.22-4.39).
Complementary feeding	Osaki, 2018<br>Cluster RCT	MCH handbook	Positive	Respondents in the intervention arm are more likely to practice complementary feeding for 6-9 months (OR=4.35, 95% CI: 2.85-6.65).
Proper feeding order	Osaki, 2018<br>Cluster RCT	MCH handbook	Positive	Respondents in the intervention arm were more likely to practice proper feeding order with breastfeeding first and complementary feeding second in 6-9 months (OR=2.70, 95% CI: 1.79-4.09).
Varied foods feeding	Osaki, 2018<br>Cluster RCT	MCH handbook	Positive	Respondents in the intervention arm were more likely to practice varied foods feeding by providing fruits and/or fruits extract (OR=2.18, 95% CI: 1.42-3.36), foods containing protein, vitamins, and oil to soft rice (OR=1.54, 95% CI: 1.03-2.30), and two snack feedings with varied food at home (OR=4.14, 95% CI: 2.70-6.34).
Self-feeding training	Osaki, 2018<br>Cluster RCT	MCH handbook	Positive	Respondents in the intervention arm were more likely to train their child to self-feed (OR=2.75, 95% CI: 1.74-4.36).
Recording immunizations	Lakhani, 1984<br>RCT	Home-based health record booklet	No impact	The proportion of booklets with entries on this page was low. The information was still being recorded on an immunization card that had been used previously.
	Mukanga, 2006<br>Cross-sectional	Child health card	Positive	Respondents whose cards were seen were more likely to record immunizations in the card (AOR=1.63, 95% CI: 0.44-3.17).
	Brown, 2018<br>Mixed methods	Home-based record	Positive	Across the 516 reviewed home-based records, recording areas were most commonly identified for the child's demographic information (80% of HBRs) and vaccination history (82%) with information marked in >90% of records.

Table S2. (continued)

Outcomes	Reference<br>Study design	Intervention	Effect of intervention	Comments
Growth monitoring	Mukanga, 2006<br>Cross-sectional	Child health card	Positive	Respondents whose cards were seen were more likely to use the card for monitoring their child’s growth (AOR=1.15, 95% CI: 0.92-2.49).
	Hamilton, 2012<br>Mixed methods	Child personal health record	No impact	Mothers did not pay attention to developmental indicators that they did not understand, such as head circumference. Further explanation may have supported mothers and reduced their concerns.
	Araujo, 2017<br>Cross-sectional	Child health handbook	No impact	High frequencies of inadequate data entry were seen, ranging from 41.1% for the weight-versus-age chart to 95.3% for the BMI-versus-age chart. Data entry was better at locations with larger healthcare teams.
**Infant and child illness management**				
Home care for cough	Osaki, 2018<br>Cluster RCT	MCH handbook	Positive	Those who had cough received home care in the intervention than control arm (OR=3.50, 95% CI: 1.44-8.52).
Home care for diarrhea	Osaki, 2018<br>Cluster RCT	MCH handbook	No impact	No significant difference between the intervention and control arm as for home care for diarrhea (83.3% of 24 cases, 92.6% of 27 cases).
Vitamin A use	Osaki, 2018<br>Cluster RCT	MCH handbook	Positive	Respondents in the intervention arm were more likely to administer vitamin A to their child (OR=2.00, 95% CI: 1.16-3.47).
	Bhuiyan, 2006<br>Mixed methods	MCH handbook	Positive	Significant change in vitamin A use was seen among mothers who received the MCH handbook compared with the control group (17.6% vs. 1.4%; *p*-value <0.05).
**Child mortality and morbidity**				
Underweight children	Osaki, 2018<br>Cluster RCT	MCH handbook	Positive	Respondents in the intervention arm were less likely to have underweight children (OR=0.33, 95% CI: 0.12-0.94).
Stunted growth	Osaki, 2018<br>Cluster RCT	MCH handbook	Positive	Respondents in the intervention arm were less likely to have stunted children (OR=0.53, 95% CI: 0.30-0.92).
Wasting	Osaki, 2018<br>Cluster RCT	MCH handbook	No impact	No significant difference between the intervention and control arm as for wasted children ((OR = 0.59, 95% CI: 0.24–1.47)
Risk of cognitive delay	Dagvadorj, 2017<br>Cluster RCT	MCH handbook	Positive	MCH handbook users showed reduction in the risk of cognitive delay compared with the control group, at a three-year follow-up (AOR=0.32, 95% CI: 0.14-0.73).
**Continuum of care**	Shah, 1993<br>Quasi-experimental	Home-based maternal record (HBMR)	Positive	The use of the HBMR had a favorable impact on utilization of health care services and continuity of the healthcare of women during their reproductive period.
	Aiga, 2016<br>Mixed methods	MCH handbook	Positive	The results of study imply that MCH Handbook contributed to the increase in pregnant women’s practices of ≥3 antenatal care visits and in their knowledge about and practice of exclusive breastfeeding. While there is room for improvement in the level of its data recording, the study confirmed that MCH Handbook plays a catalytic role in ensuring a continuum of maternal, newborn and child care.
	Kaneko, 2017<br>Quasi-experimental	MCH handbook	Positive	The proportion of mothers who received notification of birth at health facilities significantly increased (*p*-value <0.05) from 4.6% to 61.0% (95% CI: 55.9-66.2%). The proportion of mothers who received guidance on PNC from health personnel after delivery significantly increased (*p*-value <0.05) from 35.9% to 64.2% (95% CI: 59.2-69.3%)

Table S2. (continued)

Outcomes	Reference<br>Study design	Intervention	Effect of intervention	Comments
	Osaki, 2013<br>Cross-sectional	MCH handbook	Positive	National data showed that service utilization (e.g., attendance at birth, maternal care continuity, completion of child immunization) was associated with ownership of both records compared with owning a single record or none (*p*-value <0.001).
	Osaki, 2018<br>Cluster RCT	MCH handbook	Positive	Respondents in the intervention arm were more likely to receive two doses of tetanus immunization, antenatal care four times, professional assistance during child delivery and ensure that children took vitamin A supplements (OR=2.03, 95% CI: 1.19-3.47). This is followed by exclusive breastfeeding (OR=2.38, 95% CI: 1.22-4.64)), continued breastfeeding (OR=2.97, 95% CI: 1.45-6.10), and complementary feeding in 6-9 months (OR=7.13, 95% CI: 2.43-20.90).
