# Supplementary material for: Roles of the Maternal and Child Health Handbook and Other Home-Based Records on Newborn and Child Health: A Systematic Review
Source: Int J Environ Res Public Health. 2021 Jul 13;18(14):7463. doi: 10.3390/ijerph18147463 (PMC8306696; doi:10.3390/ijerph18147463)
Supplement: Supplementary file 1 [file ijerph-18-07463-s001.zip › Table S3. Japanese articles.pdf]

Table S3. Japanese articles showing the effects of the MCH handbook on newborn and child health

Outcomes	Reference<br>Study design	Intervention	Effect of intervention	Comments
**Vaccination use/uptake**				
Rotavirus vaccination	Inoue, 2015<br>Cross-sectional	MCH handbook	Mixed	The vaccination rate of Rotarix or rotaTeq was 33.4%. Most children were vaccinated in the recommmended period. The number of children vaccinated was significantly smaller with Rotarix or RotaTeq than with universal vaccines. Moreover, significantly fewer children were vaccinated against rotavirus than against Hib and varicella.
BCG and polio vaccination	Yamamoto, 1998<br>Cross-sectional	MCH handbook	Mixed	BCG and polio which are group administered at the health center have high vaccination coverage (93%). Most knowledge regarding vaccination is from the MCH handbook. There are few whom consult medical workers. One obstacle making vaccination difficult is the fact that group vaccination dates and times are fixed. For the individual administration, the difficulties of deciding the order of vaccination and concern about side effects.
Mumps, measles, chickenpox	Kimura, 2010<br>Cross-sectional	MCH handbook	Mixed	Vaccination for chicken pox and measles before elementary school was 8.5% and 13.9% respectively. For the 6th graders, vaccination coverage rates were 8.5% and 13.2% respectively for chicken pox and measles, which is much lower than the recommended 30% level.
**Vaccination history/ records**	Kanno, 1988<br>Cross-sectional	MCH handbook	Positive	DPT 1 coverage was more than 90%, DPT 2 was 82.3%, polio 1 and polio 2 were 98.0% and 95.7% respectively. A tuberculin response was detected in 95.2% (of which BCG had been administered to 86.9%), measles coverage was 92.9%, mumps was 24.8%, Japanese encephalitis 1 coverage was 64.5%, Japanese encephalitis 2 coverage was 59.8% and the booster was 42.9%. The first influenza shot was given to 65.0% coverage decreased for subsequent shots with only 3.7% of children having received the shot 3 years in a row.
	Shimizu, 2007<br>Cross-sectional	MCH handbook	Positive	The immunization coverage improved. No data was presented.
	Ogasawara, 2016<br>Cross-sectional	MCH handbook	Positive	The "vaccination record", "delivery situation", "one month check-up" and other useful information were recorded. Iwate Prefecture's perinatal medical information system "Iiha-tobu" and the MCH handbook were useful during the disaster and utilized widely. For the MCH handbook to be able to survive future large disasters, efforts must be made to realize e-MCH handbook and for data to be kept in the cloud.
**Child care-seeking**				
Use of health care and laboratory services	Nakazawa, 2007<br>Cross-sectional	MCH handbook	Positive	Thirty-five infants were diagnosed to have both ABR (Auditory Brainstem Response) and a list (high or low) score by three months of age. Twenty-one cases had normal hearing and almost full scores. Two cases of severe hearing loss had very low scores. One case with moderate hearing loss also had a very low score, whereas another had a very high score; this later case was proved to have high-frequency hearing loss.

Table S3. (continued)

Outcomes	Reference<br>Study design	Intervention	Effect of intervention	Comments
**Child health care knowledge**				
Immunization	Yahata, 2005<br>Qualitative	MCH handbook	Mixed	Caregivers were not against measles vaccination (positive attitude) The main reasons why they had not vaccinated their child against measles were "My child caught a cold, and it was difficult to find time afterwards", "I also intend to go vaccinate my child but can not seem to get there", "I don't have time to go for vaccination".
	Oguchi, 2014<br>Cross-sectional	MCH handbook	No impact	Of 556 children eligible for measles and rubella 2 vaccination, 523 children (94.1%) were vaccinated. Administrators faulted caretaker understanding and lack of information to the low coverage rate. Measles symptoms were known but some negated the need to vaccinate or even be infected with measles. Information sources were public announcements, mother, friends and the MCH handbook. Motivation to vaccinate for 365 mothers (70.0%) was the official announcement. Some mothers were concerned about vaccine side-effects.
	Kamiya, 2016<br>Cross-sectional	MCH handbook	No impact	56.9% of mothers were in their early 30s. During pregnancy, 33.3% of the mothers obtained information on when to vaccinate and the time between vaccination from explanatory documents, 32.5% of mothers from their MCH handbook. The information was easy to understand for 69.1% but 43.9% of mothers had experienced difficulties at around 2-3 months after birth. 35.0% of mothers wished to have more information during pregnancy. 56.1 % requested information on the vaccination schedule.
Detection of biliary atresia	Yamagiwa, 2009<br>Cross-sectional	MCH handbook stool card	Mixed	There were no significant differences (*p*-value =0.171) in the knowledge of the disease entity of biliary atresia between Group A (MCH handbook appending the color card) and Group B (MCH handbook without the color card). However, the knowledge about the stool color of biliary atresia (*p*-value =0.017) and the level of interest about the disease (*p*-value =0.010) were significantly higher in Group A.
	Hirayama, 2011<br>Cross-sectional	MCH handbook stool card	Positive	Before obtaining the handbook, only 77 mothers (32.2%) had heard of biliary atresia. In contrast, 137 mothers (57.7%) had increased concerns about the color card for the early diagnosis of biliary atresia when they received the handbook before delivery, and in practice, 203 mothers (84.9%) reported comparing the color of her infant's stool with this card. Moreover, four mothers reported an episode where they had noted light-colored stool of their infants, and one mother consulted a hospital on day 8 after birth. Her child was thereafter diagnosed to have biliary atresia. Finally, 236 mothers (98.7%) replied that the handbook of Niigata prefecture was useful for them and was helpful in ensuring the early diagnosis of this disease.
	Yokoi, 2019<br>Retrospective cohort	MCH handbook stool card	Mixed	Most of the survey participants (87%) considered the stool color card useful for the early detection of biliary atresia. However, they also answered that they needed more information about the card.

Table S3. (continued)

Outcomes	Reference Study design	Intervention	Effect of intervention	Comments
Sudden Infant Death Syndrome	Ichikawa, 2016 Cross-sectional	MCH handbook	Mixed	Mothers who had the knowledge of the Sudden Infant Death Syndrome (SIDS) was 118 (31.2%), the number of mothers who had only heard was 234 (61.9%). There are 15 mothers (4.0%) who didn't know SIDS at all. Sixty-six mothers replied that they "actively use the MCH handbook", 198 mothers replied that they "use the MCH handbook more or less", 93 mothers "do not actively use the MCH handbook", 19 mothers "do not use the MCH handbook", 2 mothers did not reply.
Prevention of infant accident	Nokubo, 2006 Cross-sectional	MCH handbook	Positive	A group of mothers who have ever read the MCH handbook are more careful of an accident in infancy than a group of mothers who have never read it (p-value <0.01).
**Child health care practices**				
Recording immunizations	Enokido, 1965 Cross-sectional	MCH handbook	Mixed	As infant health check-up records are filled in by public nurses, use rose from 53.1% four years ago to 77.2%. However, vaccination records were not kept well with only 30.7% taking note
	Fujimoto, 2001 Cross-sectional	MCH handbook	Mixed	High ratio of caregivers who read and wrote in the MCH handbook. Loss was minimal at 0.9%. The most responses for the most useful page was the "vaccination record". Many expected to see improvements in "parenting" information.
Growth monitoring	Enokido, 1965 Cross-sectional	MCH handbook	Mixed	About 83% of mothers had filled out the neonatal outcomes (weight and height), an increase of 30.4% compared to four years ago. However, record keeping for other neonatal outcomes were not filled in for over 55%.
	Hokama, 2000 Cross-sectional	MCH handbook	Positive	Over 70% of mothers had read the pages on parenting. More than half of the mothers had filled in the pages of their child's development and growth chart. Reading and filling out the handbook were associated with maternal characteristics, with older mothers and mothers with little childcare experience filling out the handbook more.
	Aoki, 2009 Cross-sectional	MCH handbook	Mixed	Checking of developmental milestones at various time points was frequent but recording of growth curves or observations of children was done less often.
	Shibahara, 2010 Cross-sectional	MCH handbook	No impact	Families were recording their children's growth and development, life at home or medical consultations in commercially available growth records, diaries, or blogs, but had given up recording items related to growth in Mother and Child Health Notebooks.
	Shibahara, 2010 Cross-sectional	Health and Living Log	Positive	Regarding the Health and Living Log created to support families with infants with lower limb disability living at home, families gave a high evaluation to the inclusion of information offered by health facilities and the items of Condition at Birth and Growth and Development.
	Fujii, 2020 Qualitative	MCH handbook	Positive	Mothers who had given birth to twins regarded the MCH handbook as a medical record that shows how the child is developing.
**Infant and child illness management**				
Home care for diarrhea	Shimizu, 2007 Cross-sectional	MCH handbook	Positive	Common problems such as diarrhea decreased. No data was presented.

Table S3. (continued)

Outcomes	Reference<br>Study design	Intervention	Effect of intervention	Comments
**Continuum of care**	Shimizu, 2007<br>Cross-sectional	MCH handbook	Positive	The rate of mothers receiving antenatal and postpartum care at designated clinics or hospitals increased from 13% to 40%.
	Yuge, 2010<br>Cross-sectional	MCH handbook	Positive	Average points on the whether mothers wanted to show the handbook to their children, on continuity was 4.5-4.8 points, mothers with 4 month old children had a higher continuity awareness than 3 year old children. Mothers who had seen their own handbook when younger had a higher continuity awareness than those who had not. There is a statistically significant association between those who see utility in the handbook and handing over the handbook to their children.
	Adachi, 2010<br>Cross-sectional	MCH handbook	Mixed	The revised booklet which seemed to have strengthened the collaborative interactions over hospital, local governments and schools. However, much efforts are required to change the positive usage habit of the MCH booklet in terms of self-responsibility for health.
