# Supplementary material for: Roles of the Maternal and Child Health Handbook and Other Home-Based Records on Newborn and Child Health: A Systematic Review
Source: Int J Environ Res Public Health. 2021 Jul 13;18(14):7463. doi: 10.3390/ijerph18147463 (PMC8306696; doi:10.3390/ijerph18147463)
Supplement: Supplementary file 1 [file ijerph-18-07463-s001.zip › Table S4. PICO table.pdf]

Table S4. Characteristics of included studies

Study	Study design	Study setting	Study population	Sample size	Intervention	Comparator	Reported outcomes
Abbott, 2013 Australia	Mixed methods	Aboriginal Medical Service Western Sydney (AMSWS); Jan. 2008-Sept. 2009	Children attending the AMSWS for any immunization at the scheduled dose points of 2, 4, 6, 12 and 18 months of age	505	Personalized immunization calendar showing the month and day when the child's next immuzation was due and a picture of the child was given.	Those who did not received personalized immunization calendars	The average delay in those who received a calendar at their previous visit was 0.6 months (95% CI= -0.8 to 2.6) after the due date, compared to 3.3 months (95% CI= -0.6 to 7.5) in those who did not. As for doses, 80% were on time in the group who received a calendar at the preceding immunization, 66% were on time for those who received a calendar at an earlier point and 57% of doses were on time for those who did not receive a calendar (p-value<0.0001). Interview data further supported the value and effectiveness of the calendars as both a prompt to timely immunisations and a community health education project without undue resource implications.
Adachi, 2010 Japan	Cross-sectional	Mie Prefecture	Guardians of one-year old child	115	MCH handbook	No comparison group	Most guardians were satisfied with the usability of the revised booklet. The revised booklet which seemed to have strengthened the collaborative interactions over hospital, local governments and schools. However, much efforts are required to change the positive usage habit of the MCH booklet in terms of self-responsibility for health.
Aiga, 2016 Vietnam	Mixed methods	Four provinces of Vietnam (Dien Bien, Hoa Binh, Thanh Hoa and An Giang)	Mothers of children 6-18 months of age in the four provinces	810	An MCH Handbook was handed to each pregnant woman being followed by face-to-face verbal guidance by a health worker upon the initial antenatal care visit.	No comparison group	The results of study imply that MCH Handbook contributed to the increase in pregnant women's practices of ≥3 antenatal care visits and in their knowledge about and practice of exclusive breastfeeding. While there is room for improvement in the level of its data recording, the study confirmed that MCH Handbook plays a catalytic role in ensuring a continuum of maternal, newborn and child care.
Aoki, 2009 Japan	Cross-sectional	Three nursery schools in Tokyo and one nursery school in Saitama Prefecture	Parents of nursery school students (0-5 years old)	298	MCH handbook	No comparison group	Checking of developmental milestones at various time points was frequent, but recording of growth curves or observations of children was done less often. Lnformation in the MCH handbook was not used frequently. In general, guardians used the handbook passively rather than actively, and only about half regarded the handbook as user-friendly. To improve the quality of the MCH handbook, guardians requested more information on child health, such as first aid, the timing of immunization, or weaning foods. On the basis of categorical data analysis of the results, a "user-friendly MCH handbook" was considered to incorporate the following points: an appropriate size, easy-to-understand expressions, and a higher content of information relevant to guardians.
Araujo, 2017 Brazil	Cross-sectional	Two municipalities in Paraiba, Brazil	Children under 5	321	Child health handbook	No comparison group	All the parameters studied showed high frequencies of inadequate data entry, ranging from 41.1% for the weight-versus- age chart to 95.3% for the body mass index-versus-age chart. Higher frequency of inadequate data entry was found among children aged 25 months and over and among those living in areas of these municipalities with minimal numbers of professionals in the healthcare teams.

Table S4. (continued)

Study	Study design	Study setting	Study population	Sample size	Intervention	Comparator	Reported outcomes
Bhuiyan, 2006 Bangladesh	Mixed methods	Maternal and Child Health Training Institute in Dhaka, Bangladesh	Pregnant women	600	MCH handbook	Standard cards	Findings from the focus group discussions emphasized the need for including MCH handbook in maternal and child program in Bangladesh. In addition, quantitative data suggests that mothers in study group had higher knowledge on MCH issues, better practices in MCH care, and higher utilization of MCH services than mothers in control groups who used other health cards.
Brown, 2018 Kenya	Mixed methods	40 health facilities across 10 counties in Kenya (4 health facilities per county)	Caregivers of children aged <24 months attending a health facility during November 2016 as well as health staff and facility administrators	677	Home-based record (HBR)	No comparison group	Of 677 children, 76% currently owned an HBR and brought the document with them to the clinic. Across the 516 reviewed HBRs, recording areas were most commonly identified for the child's demographic information (80% of HBRs) and vaccination history (82%) with information marked in >90% of records. Recording areas were less frequently available for child early eye / vision problems (61%), growth monitoring (74%) and vitamin A (76%); with information marked in 33%, 88% and 60% of records, respectively.
Dagvadorj, 2017 Mongolia	Cluster RCT	Bulgan province of Mongolia	All women living in the Bulgan Province of Mongolia who gave birth between March and August 2010	337	MCH handbook	Non-user of MCH handbook	Distribution of the handbook had a protective effect on the risk of cognitive delay, but poor cognitive development was increased by higher maternal age, maternal depression one month after delivery and pregnancy complications. Actively using the MCH handbook for three years helped to lower the risk of impaired cognitive development, specifically by recording infant check-ups and growth curves and reading the sections on antenatal and delivery records and developmental milestones.
Enokido, 1964 Japan	Cross-sectional	Ryugasaki village and six other neighboring villages in Ibaraki Prefecture	3 year olds and infants	989	MCH handbook	No comparison group	Four years ago, basic information on the mother and child (name, address) was filled in by only 59.3%. The present study finds 77.2% have filled in the page. Birth registration certification page was filled in for 88.4%, an increase of 10.5% in two villages. About 82.8% of mothers had filled out the neonatal outcomes (weight and height), an increase of 30.4% compared to four years ago. However, record keeping for other neonatal outcomes were not filled in for over 55%. As infant health check-up records are filled in by public nurses, use rose from 53.1% four years ago to 77.2%. Vaccination records however were not kept well with only 30.7% taking note.
Fujii, 2020 Japan	Qualitative	Support group for mothers who gave birth to twins; near Tokyo city	Mothers who gave birth to twins in one or two years prior to study	5	MCH handbook	No comparison group	Mothers who had given birth to twins, regarded the handbook as "evidence of their readiness to become mothers of twins," "hope of becoming a good mother," "something that should prevent anxiety related to having a high-risk pregnancy," "a medical record that shows how the child is developing" and "they stopped using the handbook on their own."

Table S4. (continued)

Study	Study design	Study setting	Study population	Sample size	Intervention	Comparator	Reported outcomes
Fujimoto, 2001 Japan	Cross-sectional	231 local towns and wards in Niigata Prefecture, Yokohama Prefecture, Shizuoka Prefecture and Hiroshima Prefecture	Caregivers who have come with their 18-month old child for 18-month check-up	10,900	MCH handbook	No comparison group	High ratio of caregivers who read and wrote in the MCH handbook. Loss was minimal at 0.9%. The most responses for the most useful page was the "vaccination record". Many expected to see improvements in "child rearing" information. Many caregivers replied in neutral when asked about the usefulness of the handbook. Oral hygiene was the least filled-out and there was only a minimum of people who replied that this page was useful.
Grøvdal, 2006 Norway	RCT	Maternal and child health centers in 10 municipalities in Norway	Parents of 309 children attending the National Preschool Health Surveillance Programme	309	Half of the parents were given a parent-held child health record (PHCHR) and short instructions on how it was expected to be used.	Parents and children who did not use PHCHR, just ordinary national health surveillance program	Some 73% of the intervention group used the PHCHR regularly when visiting the health centers, 79% reported that their own writing in the record was helpful, and 92% favored the PHCHR being permanently adopted. Use of the record did not influence the utilization of healthcare services, parents' knowledge of their child's health, or parents' satisfaction with information or communication with professionals.
Haeri Mazanderani, 2018 South Africa	Retrospective cohort	Tshwane District Clinical Services	Newborn infants regardless of nationality or HIV exposure status between May 2016 and May 2017	309	A sticker-page of unique, readable, barcoded patient identifiers was incorporated in the patient-retained immunization record of the Road-to-Health-Booklet (RTHB) before distribution.	No comparison group	The number of registered RTHB identifiers increased from 24 (2% of birth PCR tests) in May 2016, peaking at 728 (56% of birth PCR tests) in May 2017. Among infants with a registered RTHB identifier at birth, 635 (12%) had a subsequent linked HIV PCR test, as indicated by the same RTHB number registered for a later specimen. Leveraging RTHBs as unique patient identifiers, even if used temporarily until linkage to other future national unique identifiers, promises to be an effective, scalable approach to laboratory-based surveillance, facilitating healthcare provider access to all test results from birth.
Hagiwara, 2013 Palestine	Quasi-experimental	MCH treatment centers	Mothers who were expose and not exposed to the MCH Handbook	340	MCH handbook	Mothers who did not use the MCH Handbook	Knowledge related to MCH such as the importance of exclusive breastfeeding and how to cope with the risks of rupture of membranes during pregnancy increased among MCH handbook users, especially among less-educated women. The MCH handbook may be an effective tool for communication with health providers and husbands, for both highly educated and less-educated women during their first pregnancy.

Table S4. (continued)

Study	Study design	Study setting	Study population	Sample size	Intervention	Comparator	Reported outcomes
Hamilton, 2012 Australia	Mixed methods	New South Wales (NSW)	Parents (mothers) who had at least one child aged between 0-4 years old	126	Child personal health record (CPHR)	No comparison group	CPHR can play an important role in communicating information regarding a child's health and development between parents and professionals, it is perhaps underutilised. Opportunities for use were reduced where there were dual systems in place, such as online records for immunization. Some information in the CPHR had the potential to escalate concerns about infant development. This was particularly the case for the growth charts, and it appeared that further explanation may have supported mothers and reduced their concerns. It was also the case that mothers did not pay attention to developmental indicators that they did not understand, such as head circumference.
Hirayama, 2011 Japan	Cross-sectional	Niigata Prefecture	Mothers who visited the hospital for their infant's one-month health check	239	MCH handbook stool card	No comparison group	Before obtaining the handbook, only 77 mothers (32.2%) had heard of biliary atresia. In contrast, 137 mothers (57.7%) had increased concerns about the color card for the early diagnosis of biliary atresia when they received the handbook before delivery, and in practice, 203 mothers (84.9%) reported comparing the color of her infant's stool with this card. Moreover, four mothers reported an episode where they had noted light-colored stool of their infants, and one mother consulted a hospital on day 8 after birth. Her child was thereafter diagnosed to have biliary atresia. Finally, 236 mothers (98.7%) replied that the handbook of Niigata prefecture was useful for them and was helpful in ensuring the early diagnosis of this disease.
Hokama, 2000, Japan	Cross-sectional	Naha, Okinawa	Mothers of 3-5 month old children who have come for check-up	281	MCH handbook	No comparison group	Over 70% of mothers had read the pages on parenting. More than half of the mothers had filled in the pages of their child's development and growth chart. Reading and filling out the handbook were associated with maternal characteristics, with older mothers and mothers with little childcare experience filling out the handbook more. Over 90% of mothers replied that the information in the handbook was useful. The most highly evaluated pages were those on child health, growth and vaccination.

Table S4. (continued)

Study	Study design	Study setting	Study population	Sample size	Intervention	Comparator	Reported outcomes
Ichikawa, 2016 Japan	Cross-sectional	Hokkaido	Mothers who brought their one-month old for check-up	378	MCH handbook	No comparison group	Mothers who had the knowledge of the Sudden Infant Death Syndrome (SIDS) was 118 (31.2%), the number of mothers who had only heard was 234 (61.9%). There are 15 mothers (4.0%) who didn’t know SIDS at all. Participants were asked three sentences of risk factors which leads to SIDS. There were 237(62.7%) mothers who wrote “lying face down” in the questionnaire, and 224(59.3%) mothers who wrote “smoking”. And there were 53(14.0%) mothers who wrote “non breast feeding”. Mothers who wrote the three sentences perfectly were only 46 (12.2%), mothers who answered two sentences were 141 (37.3%), and who wrote one only one sentence were 94 (24.9%). The mothers who wrote nothing were 97 (25.7%). In all, 66 mothers replied that they “actively use the MCH handbook,” 198 mothers replied that they “use the MCH handbook more or less,” 93 mothers “do not actively use the MCH handbook,” 19 mothers “do not use the MCH handbook,” 2 mothers did not reply.
Inoue, 2015 Japan	Cross-sectional	Fuchu-shi Tokyo, Japan	18 month old children	376	MCH handbook	No comparison group	The vaccination rate of Rotarix or ToaTeq was 33.4% (95% CI: 4.6%). Most children were vaccinated in the recommmended period. The number of children vaccinated was significantly smaller with Rotarix or RotaTeq than with universal vaccines. Moreover, significantly fewer children were vaccinated against rotavirus than against Hib and varicella.
Jeffs, 1994 Australia	Quasi-experimental	New South Wales (NSW), Australia	Households with children aged four years or less and health care providers	1,533	Introduction of personal health records (PHR) since 1988	Five years after the introduction of personal health records	PHR was well retained, with 89% claimed retention at 4 years, and over 78% of parents able to produce the record for inspection at interview. Of the records examined, 91% had at least one immunization recorded while 68% had a complete regimen documented by age 4 years. Overall, 93% of parents expressed satisfaction with the PHR, while 64% of all health care providers also felt that the PHR was ‘beneficial to the health care children received’, although only 53% of them used it regularly to record their findings. It is concluded that the PHR currently issued in NSW is well retained and valued by parents, and used by and useful to a range of health professionals.
Kamiya, 2016 Japan	Cross-sectional	Pre-school children parenting space	Mothers who use the pre-school children parenting space	123	MCH handbook	No comparison group	More than half (56.9%) of mothers were in their early 30s. During pregnancy, 33.3% of the mothers obtained information on when to vaccinate and the time between vacination from explanatory documents, 32.5% of mothers from their MCH handbook. The information was easy to understand for 69.1% but 43.9% of mothers had experienced difficulties at around 2-3 months after birth. About 35.0% of mothers wished to have more information during pregnancy and 56.1 % requested information on the vaccination schedule.

Table S4. (continued)

Study	Study design	Study setting	Study population	Sample size	Intervention	Comparator	Reported outcomes
Kaneko, 2017 Burundi	Quasi-experimental	Gitega District,23 health facilities providing maternal and child health care services in the Gitega District	Mothers with infants aged less than six weeks and living within the jurisdiction of each health facility	344	MCH handbook	No comparison group	Majority of the mothers (95.1%) had an MCH handbook post-study. Significant improvement was observed in the proportion of mothers receiving notification of birth at health facilities, from 4.6% to 61.0% (95% confidence interval [CI]= 55.9%–66.2%), and the proportion of mothers receiving guidance on PNC, from 35.9% to 64.2% (95% CI= 59.2%–69.3%). As previous studies showed, the MCH handbook appeared to help health personnel provide guidance on PNC, thereby it may have increased PNC. Furthermore, this study suggests the handbook contributed to every birth being counted. However, to increase the effectiveness of the handbook, health personnel should be encouraged toward its proper use.
Kanno, 1988 Japan	Cross-sectional	Primary school registration	Children registering for primary school	651	MCH handbook	No comparison group	DPT 1 coverage was more than 90%, DPT 2 was 82.3%, polio 1 and polio 2 were 98.0% and 95.7% respectively. A tuberculin response was detected in 95.2% (of which BCG had been administered to 86.9%), measles coverage was 92.9%, mumps was 24.8%, Japanese encephalitis 1 coverage was 64.5%, Japanese encephalitis 2 coverage was 59.8% and the booster was 42.9%. The first influenza shot was given to 65.0% coverage decreased for subsequent shots with only 3.7% of children having received the shot 3 years in a row. When looking at age when each vaccine was administered, DPT was administered between 13-24 months, with some having received the shots between 7 to 12 months. Polio was second and administered by 6 months. Tuberculin response was seen at the second polio vaccination. Measles vaccine were administered mostly between 13-24 months, with a minority having done it during 7- 12 months. Mumps was mostly administered after 2 years and was not administered to under one-year old children.
Kawakatsu, 2015 Kenya	Cross-sectional	Rural western Kenya	Mothers with children aged 12-24 months	2,560	MCH handbook	No comparison group	Impacts of 5.9, 9.4, and 12.6 percentage points for higher health knowledge and for proper health-seeking behavior for fever and diarrhea, respectively, were statistically significant.The significant factors affecting possession of the MCH Handbook were the child's sex, the caregiver's relationship to the child, maternal age, health knowledge, birth interval, household wealth index and community health worker performance accordingly. An MCH handbook was an effective tool for improving both health knowledge and health-seeking behavior in Kenya. The further distribution and utilization of an MCH handbook is expected to be an effective way to improve both maternal and child health.

Table S4. (continued)

Study	Study design	Study setting	Study population	Sample size	Intervention	Comparator	Reported outcomes
Kimura, 2010 Japan	Cross-sectional	Private clinic in Shimane Prefecture, Japan	Pre-schoolers and 6th graders	329	MCH handbook	No comparison group	For the 223 pre-elementary school aged children, 5.8% were susceptible for chicken pox and 43.5% for mumps. For the 106 Grade 6 students, 2.8% were susceptible for chicken pox and 30.2% for mumps. Vaccination for chicken pox and measles before elementary school was 8.5% and 13.9% respectively. For the 6th graders, vaccination coverage rates were 8.5% and 13.2% respectively for chicken pox and measles, which is much lower than the recommended 30% level.
Kreuter, 2004 USA	Quasi-experimental	Two neighborhoods in St. Louis, Missouri	Parents of babies aged birth to one year who visited the pediatrics department at two public health centers	321	Individually tailored immunization calendar	Those who did not receive the calendar	A higher proportion of intervention than of control babies were up to date at the end of a 9-month enrollment period (82% vs 65%, *p*-value<0.001) and at age 24 months (66% vs 47%, *p*-value<0.001). The younger the baby's age at enrollment in the program, the greater was the intervention effect. Tailored immunization calendars can help increase child immunization rates.
Lakhani, 1984 UK	RCT	St Thomas hospital	Mothers resident in the West Lambeth Health District and discharged from obstetric wards at St Thomas's hospital between May and August 1980.	479	At discharge, mothers on study wards were given a booklet in a sealed envelope and asked to discuss its use with health visitors.	No intervention for the period of May to August 1980. In September 1980, it had been decided that booklets would be distributed to all mothers in the district.	There was no significant difference in the uptake of immunization and developmental assessment clinic attendance between the study and control groups. Mothers whose first language was English made more developmental milestone entries.
Lovell, 1987 UK	RCT	Antenatal clinic in St. Thomas's Hospital	Expectant mothers	246	Maternity case notes	Standard cooperation card	More of the notes group expressed satisfaction with most aspects of their care and delivery and significantly more of the notes group felt well informed and satisfied with their companion during labor. There were no differences in clinical outcomes between the two groups, except that there were more assisted deliveries among the notes group for no identifiable systematic reason.
McElligott, 2010 USA	Cross-sectional	Households	National Immunization Survery (NIS) from 2004 to 2006, a national, validated survey of households with children 19 to 35 months of age	5,940,204	Patient-held vaccination records	Children without vaccination records	Children with vaccination records were more likely to be up-to-date (83.9% vs 78.6%; *p*-value<0.001). Vaccination record was associated with a 62% increase in the odds of up-to-date status. The largest effects associated with vaccination records were seen for children with multiple providers, comparing with and without vaccination record (82.8% vs 71.9%; *p*-value<0.001), those with low maternal education, (81.6% vs 72.9%; *p*-value<0.001), and those with >= 4 children in the household (76% vs 69.6%; *p*-value<0.004).

Table S4. (continued)

Study	Study design	Study setting	Study population	Sample size	Intervention	Comparator	Reported outcomes
McMaster, 1996 Bosnia and Herzegovina	Cross-sectional	Near Tuzla	Mothers and children in the collective centers and from the local community	571	Booklets (incorporating health records and health advice) were distributed to displaced and other families	No comparison group	Personal child health record and advice booklets not only provided essential data on immunization, nutrition, and prevalent medical disorders but also appeared to benefit the young population by supplying a permanent health record and health education material.
Moore, 2000 UK	Quasi-experimental	Leicestershire county	Parents of British children who are likely to have special educational needs	99	Designed a record for disabled children as a supplement to the Leicestershire child health record. The intervention phase lasted 6 months. Only families in groups 1 and 3 received the new record.	Families who did not use the new record (Group 2)	Most of the entries were factual, and the principal use of the new document was as an aide-memoire. There was no evidence that the record improved the parent's perception of their child's general health care, nor that it contributed to the overall level of communication between parents and professionals.
Mori, 2015 Mongolia	Cluster RCT	18 units (16 soums and 2 bags) in Bulgan, Mongolia	Pregnant women and their infants living in Bulgan, Mongolia from May 2009 to September 2010	501	The MCH handbook was implemented immediately for women at their first antenatal visit in the intervention group, and nine months later in the control group.	No intervention for the period of the original trial.	Women of higher socioeconomic status visited antenatal care clinics more often. Pregnancy complications were more likely to be detected among women using the handbook. There is no significant difference in early breastfeeding initiation between the intervention and control group. The majority of women stopped drinking alcohol during pregnancy.
Mudany, 2015 Kenya	Cross-sectional	Pilot phase to health facilities in Nyanza, Kenya from August 2007 to July 2008. After final revision, distributed nationally in April 2010.	Pregnant women visiting the antenatal clinic	1.5 M booklets were distributed nationally each year from 2010	Mother and Child Health booklet was distributed to every pregnant women visiting the antenatal clinic in April 2010.	During the pilot phase, seven provinces did not receive the booklet.	During the pilot period, the number of infants tested for HIV DNA increased in Nyanza from 9,966 to 13,379, a 34% increase compared with 9% overall increment in the remaining seven provinces where the booklet was not introduced. After final revision and national distribution, HIV DNA testing in infants rose from 55,000 in 2010 to 60,000 in 2012, which represents approximately 60% coverage of estimated HIV-exposed infants.
Mukanga, 2006 Uganda	Cross-sectional	Mulago II Parish	Children up to two years of age and their mothers/ caretakers	260	Child health cards	No comparison group	Child heath cards help health workers and caretakers follow up child health issues, inherently promoting child health. Children who had cards were 10 times more likely to be up to date with the immunization schedule (OR=9.55; 95% CI= 3.19-29.45). The factors associated with card retention include whether the mother or child used a formal facility where cards are issued.

Table S4. (continued)

Study	Study design	Study setting	Study population	Sample size	Intervention	Comparator	Reported outcomes
Nakazawa, 2007 Japan	Cross-sectional	Akita Prefecture, Japan	Infants who underwent hearing screening and secondary testing between 2001-2006 based on the MCH handbook Hearing Development checklist at Nakadohri Hospitial	35	MCH handbook hearing development checklist	No comparison group	35 babies were diagnosed to have both ABR (Auditory Brainstem Response) and a list (high or low) score by three months of age. Twenty-one cases had normal hearing and almost full scores. Two cases of severe hearing loss had very low scores. One case with moderate hearing loss also had a very low score, whereas another had a very high score; this later case was proved to have high-frenquency hearing loss. A girl with 80dB in the right ear and 50dB in the left ear scored alomost perfectly by the list (rarely seen). A girl with mild loss on the list scored perfectly. The rest of the cases were dropouts or little information could be obtained due to deafness in the family. The conclusions are that even if a baby scores just a few points on the list, the family should be advised to get his/her hearing checked further. The difficulities in checking babies for hearing impairments, including high-frequency loss and mild to severe hearing loss should be noted.
Nasir, 2017 Indonesia	Quasi-experimental	Tangerang Selatan District, an urban area in Banten Province	Mothers with newborns	427	MCH handbook was used as the main reference material during the mother class	Usual care	Mother class used Maternal and Child Health Handbook as the main reference material. Attending mother class significantly increased knowledge of breastfeeding initiation and hepatitis B immunization (*p*-value<0.05). Mothers in the intervention group had the likelihood of practicing good newborn care compared with the control group (OR: 1.812; 95% confidence interval: 1.235–2.660). Mother class improves knowledge and newborn care practices and strengthens interactions between mothers and health care providers.
Nokubo, 2006 Japan	Cross-sectional	Public health center, Mie prefecture	Mothers whose children have a routine health examination	69	MCH handbook	Mothers who have not read contents of MCH Handbook	A group of mothers who have ever read Maternal and Child Health Handbook is more careful of an accident in infancy than a group of mothers who have never read it.
Ogasawara, 2016 Japan	Cross-sectional	Great East Japan Earthquake disaster areas	Mothers, health and medical staff working in the disaster area	51	MCH handbook	No comparison group	The “vaccination record”, “delivery situation”, “1 month check-up” and other useful information were recorded. Iwate Prefecture’s perinatal medical information system “Iiha-tobu” and the MCH handbook were useful during the disaster and utilized widely. For the MCH handbook to be able to survive future large disasters, efforts must be made to realize e-MCH handbook and for data to be kept in the cloud.

Table S4. (continued)

Study	Study design	Study setting	Study population	Sample size	Intervention	Comparator	Reported outcomes
Oguchi, 2014 Japan	Cross-sectional	A prefecture administration and nursery school	Local administrators and mothers of children aged 5 and over	774	MCH handbook	No comparison group	Of 556 children eligible for measles and rubella 2 vaccination, 523 children (94.1%) were vaccinated. Administrators faulted caretaker understanding and lack of information to the low coverage rate. Measles symptoms were known but some negated the need to vaccinate or even be infected with measles. Information sources were public announcements, mother, friends and the MCH handbook. Motivation to vaccinate for 365 mothers (70.0%) was the official announcement. Some mothers were concerned about vaccine side-effects.
Osaki, 2013 Indonesia	Cross-sectional	Both national and provincial data from West Sumatra and North Sulawesi	Mothers	17,363	Home-based records	Non-home-based record owners	Pre- and post-natal record ownership increased from 1997 to 2007. National data showed that service utilization was associated with ownership of both records compared with owning a single record or none. These results suggest that pre- and post-natal home-based record use may be effective for ensuring service utilization. It could also be an effective tool for promoting the continuum of MNCH care in Indonesia.
Osaki, 2018 Indonesia	Cluster RCT	13 health centers in Garut district of rural Java, Indonesia	Pregnant women attending one of the selected health centers between 2007 and 2009	454	MCH handbook	Usual care	Respondents in the intervention area received consecutive MCH services including two doses of tetanus toxoid injections and antenatal care four times or more during pregnancy, professional assistance during child delivery and vitamin A supplements administration to their children, after adjustment for confounding variables and cluster effects (OR=2.03, 95% CI: 1.19–3.47). In the intervention area, home care (continued breastfeeding; introducing complementary feeding; proper feeding order; varied foods feeding; self-feeding training; and care for cough), perceived support by husbands, and lower underweight rates and stunting rates among children were observed.
Shah, 1993 Multi-countries	Quasi-experimental	13 centers in eight countries (Egypt, India, Pakistan, Philippines, Senegal, Sri Lanka, Democratic Yemen, and Zambia)	The participating centers tested the HBMR in a variety of circumstances, such as literate and illiterate populations, different geographical and cultural conditions, and communities with easy or poor access to health services in rural and urban populations.	14,000 to 250,000	Home-based maternal record (HBMR)	Non-user of HBMR	The used of the HBMR had a favorable impact on utilization of health care services and continuity of the health care of women during their reproductive period. When adapted to local risk conditions, the HBMR succeeded in promoting self-care by mothers and their families. The introduction of the HBMR increased the diagnosis and referral of at-risk pregnant women and newborn infants, improved family planning and health education, led to an increase in tetanus toxoid immunization, and provided a means of collecting health information in the community. The HBMR was liked by mothers, community health workers and other health care personnel. Mothers became more involved in looking after their own health and that of their babies. The training and involvement of health personnel from the start of the HBMR scheme influenced its success in promoting maternal and child health care. It also improved the collection of community-based data and the linking of referral networks.

Table S4. (continued)

Study	Study design	Study setting	Study population	Sample size	Intervention	Comparator	Reported outcomes
Shibahara, 2010 Japan	Cross-sectional	Kindergarten facility for infants with physical disabilities in T city	Families with children attending a kindergarten facility for infants with physical disabilities in T city	44	MCH handbook and health and living log	No comparison group	Families were recording their children's growth and development, life at home or medical consultations in commercially available growth records, diaries, or blogs, but had given up recording items related to growth in Mother and Child Health Notebooks. Regarding the Health and Living Log, families gave a high evaluation to the inclusion of information offered by health facilities and the items of Condition at Birth and Growth and Development.
Shimizu, 2007 Dominican Republic	Cross-sectional	Dajabón	Mothers who received the MCH Handbook and children under the age of 5 using the handbook	6,633	MCH handbook	No comparison group	The evaluation and regular monitoring visits revealed positive results: as for pregnant women, the handbooks were well accepted for their friendliness, simplicity, durability and mobility, and the rate of their receiving antenatal and postpartum cares at designated clinics or hospitals increased; as for newborns and children, the immunization coverage improved while common problems such as diarrhea decreased; and as for health personnel, the handbook helped clarify the division of work and enhanced their sense of responsibility, communication, and continuity and integration of service.
Stille, 2001 USA	RCT	Pediatric primary care sites in Hartford, Connecticut	Infants born at any hospital in Hartford between October 1997 and May 1998.	315	Interactive graphic card with verbal reinforcement	Routine information	After the trial, age-appropriate immunization rates at seven months were 58% in each group. Intervention infants had 50% fewer missed opportunities to immunize (*p*-value=0.01) but cancelled 77% more appointments (*p*-value=0.04) than controls. The brief educational intervention at the first well-child care visit did not boost seven-month immunization rates, although it was associated with fewer missed opportunities to immunize.
Tom, 2014 USA	Retrospective cohort	Kaiser Permanente (KP) Hawaii and Northwest regions	Children who were KP members as of July 31, 2011	10,136	Personal health record (PHR)	Non-user of PHR	Children whose parents used >= 1 PHR feature (vs none) had higher odds of adhering to the recommended immunizations only at KP Northwest (KP Hawaii: OR=1.1, 95% CI=0.8-1.4, *p*-value >0.05; KP Northwest: OR=1.2, 95% CI=1.0-1.3, *p*-value<0.05). PHR use was associated with better adherence to well-child care (WCC) visit recommendations for both KP Hawaii (OR=1.9, 95% CI=1.3-2.9, *p*-value<0.001) and KP Northwest (OR=2.5, 95% CI=2.1-2.9, *p*-value<0.001). Young children whose parents used a PHR were more likely to adhere to the recommended WCC visits in both regions but immunizations in only one region.
Usman, 2009 Pakistan	RCT	Expanded Programme on Immunization (EPI) centers located in urban areas of Karachi city	All children visiting the selected EPI centers for the first dose of diptheria-pertussis-tetanus (DPT1)	1,500	Redesigned card (Group 1), center-based education (Group 2), and redesigned card with center-based education (Group 3)	Standard care (Group 4)	A significant increase of 31% (adjusted risk ratio [RR]=1.31, 95% CI: 1.18-1.46) in DPT3 completion was estimated in the group that received both redesigned card and center-based education compared with the standard care group.

Table S4. (continued)

Study	Study design	Study setting	Study population	Sample size	Intervention	Comparator	Reported outcomes
Usman, 2011 Pakistan	RCT	Six EPI centres located in the rural peripheries of Karachi, the capital city of Sindh Province	All children visiting the selected EPI centres for DPT1 immunization	1506	Redesigned card (Group 1), center-based education (Group 2), and redesigned card with center-based education (Group 3)	Standard care (Group 4)	By the end of follow-up, 39% of children in standard care group completed DTP3. Compared to this, a significantly higher proportion of children completed DTP3 in redesigned card group (66%) (crude risk ratio [RR] = 1.7; 95% CI = 1.5, 2.0), centre-based education group (61%) (RR = 1.5; 95% CI = 1.3, 1.8) and combined intervention group (67%) (RR = 1.7; 95% CI = 1.4, 2.0). Improved immunization card alone, education to mothers alone, or both together were all effective in increasing follow-up immunization visits.
Yahata, 2005 Japan	Qualitative	Akita prefecture	Parents of non-measles vaccinated children	9	MCH handbook	No comparison group	Caregivers were not against measles vaccination (positive attitude) The main reasons why they had not vaccinated their child against measles were "My child caught a cold, and it was difficult to find time afterwards", "I also intend to go vaccinate my child but can not seem to get there", "I don't have time to go for vaccination". In order to raise vaccination coverage rate, caregivers proposed clearer messaging on "measles vaccination safety in the MCH handbook" and information that "Vaccination can be done even outside your local burrough", or other information such as "If measles vaccination dates were fixed, I would do everything to get my child vaccinated then".Others also said that the health administrators should play a more active role such as "Getting health workers to flag that measles vaccination has not been done at child health days".
Yamagiwa, 2009 Japan	Cross-sectional	Obstetrics clinic, Niigata City	Pregnant women	117	MCH handbook with stool card	Those who received the MCH Handbook without stool card	Although there were no significant differences in the knowledge about the disease entity of biliary atresia, the knowledge about the stool color of biliary atresia and the level of interest about the disease was significantly higher in the group who received MCH Handbook with stool card.
Yamamoto, 1998 Japan	Cross-sectional	Four public nurseries, one public kindergarten, mother's class for three-year-old children	Mothers of children between 1 year to 6.5 years	302	MCH handbook	No comparison group	BCG and polio which are group administered at the health center have high vaccination coverage (93%).Vaccines which are administered individually have lower coverage due to concerns about side effects and the lack of awareness of the necessity of vaccination. Most knowledge regarding vaccination is from the MCH handbook. There are few whom consult medical workers. One obstacle making vaccination difficult is the fact that group vaccination dates and times are fixed. For the individual administration, the difficulties of deciding the order of vaccination and concern about side effects.

Table S4. (continued)

Study	Study design	Study setting	Study population	Sample size	Intervention	Comparator	Reported outcomes
Yanagisawa, 2015 Cambodia	RCT	Two districts in Kampong Cham Province, Cambodia	Women who had given birth within 1 year before the survey	640	MCH Handbook and health education using the handbook	Use of child growth card and Mother Heath Record	The intervention increased maternal knowledge of all topics addressed except for the risk of severe bleeding after delivery. Logistic regression showed that the intervention increased ANC attendance, delivery with SBAs, and delivery at a health facility, even after adjusting for maternal age, education, and economic conditions. The qualitative data indicated that the handbook was well received and culturally appropriate. Thus, the MCH handbook is a reasonable and superior alternative to current card-type maternal records.
Yokoi, 2019 Japan	Retrospective cohort	Kobe Children's Hospital, Kobe City	Patients who underwent Kasai enterostomy (KPE) at the hospital from 2005 to 2018	41	MCH Handbook stool color card (SCC)	Non-user of MCH Handbook stool color card	Most of the survey participants (87%) considered the stool color card useful for the early detection of biliary atresia. However, they also answered that they needed more information about the card. The usefulness of SCC for the early detection of biliary atresia might be limited, unless parents, as well as health care providers, are more informed about SCC.
Yuge, 2010 Japan	Cross-sectional	Health check-up stations	Mothers of four-month-old, 18-month old and three-year old children who have come for check-up	321	MCH handbook	No comparison group	Utility point average was 3.4-3.5. There was no difference between child age and mother and child health status. Mothers found the pages which medical workers filled out useful. These were "delivery record", "vaccination record" and "neonatal record" pages. There were very few childcare instruction items/pages which were useful. Mothers with previous children found the pages "experience of seeing the MCH handbook during childhood", "discuss the handbook", "received explanations from the pediatrician using the handbook" more useful than first-time mothers. Average points on the whether mothers wanted to show the handbook to their children, on continuity was 4.5-4.8 points, mothers with 4 month old children had a higher continuity awareness than 3 year old children. Mothers who had seen their own handbook when younger had a higher continuity awareness than those who had not. There is a statistically significant association between those who see utility in the handbook and handing over the handbook to their children.
Zhou, 2015 USA	Retrospective cohort	Kaiser Permanente Hawaii and Northwest Regions in 2007-2011	Children who were Kaiser Permanente members	2,286	Personal health record (PHR)	Non-user of PHR	PHR-registered children, compared with propensity score-matched nonregistered children, had 21% (95% CI=14-28; *p*-value<0.001) more outpatient clinic visits and 26% (95% CI=16-37; *p*-value <0.001) more telephone encounters. Utilization differences were more pronounced with nonprimary care providers than with primary care providers.
