# Supplementary material for: Roles of the Maternal and Child Health Handbook and Other Home-Based Records on Newborn and Child Health: A Systematic Review
Source: Int J Environ Res Public Health. 2021 Jul 13;18(14):7463. doi: 10.3390/ijerph18147463 (PMC8306696; doi:10.3390/ijerph18147463)
Supplement: Supplementary file 1 [file ijerph-18-07463-s001.zip › Table S5. Risk of Bias.pdf]

Table S5. Risk of bias for observational studies

Risk of bias assessment of quasi-experimental studies
(Please indicate whether low, moderate, serious, critical, no information)

Author	Selection of participants	Confounding variables	Classification of interventions	Deviations from intended interventions	Missing data	Measurement of the outcome	Selection of the reported result	Overall risk of bias
Hagiwara 2013	Low	Moderate	Low	Moderate	Low	Low	Low	Moderate
Jeffs 1994	Low	Moderate	Low	Moderate	Low	Low	Low	Moderate
Kaneko 2017	Low	Low	Low	Moderate	Low	Low	Low	Moderate
Kreuter 2004	Low	Moderate	Low	Moderate	Low	Low	Low	Moderate
Moore 2000	Low	Moderate	Low	Serious	Moderate	Moderate	Moderate	Serious
Nasir 2017	Low	Moderate	Low	Moderate	Low	Low	Low	Moderate
Shah 1993	Low	Serious	Moderate	Moderate	Serious	Serious	Serious	Serious

Risk of bias assessment of observational cohort and cross-sectional studies
(Please indicate whether yes, no, CD [cannot determine], NA [not applicable], NR [not reported])

Author	1	2	3	4	5	6	7	8	9	10	11	12	13	14
Adachi 2010	Yes	Yes	No	Yes	No	No	CD	No	Yes	No	Yes	NA	NA	No
Aoki 2009	Yes	Yes	Yes	Yes	No	Yes	No	No	No	No	Yes	NA	NA	No
Araujo 2017	Yes	Yes	No	Yes	No	Yes	Yes	Yes	Yes	No	Yes	NR	NA	Yes
Enokido 1964	Yes	Yes	CD	Yes	No	No	Yes	NA	Yes	No	Yes	NA	NA	No
Fujimoto 2001	Yes	Yes	Yes	Yes	Yes	No	Yes	No	No	No	Yes	No	No	No
Haeri Mazanderani 2018	Yes	Yes	CD	Yes	NA	Yes	Yes	NA	Yes	Yes	Yes	CD	NR	No
Hirayama 2011	Yes	Yes	CD	Yes	No	No	Yes	No	Yes	No	Yes	No	NA	No
Hokama 2000	Yes	Yes	CD	Yes	No	No	Yes	No	Yes	No	Yes	NA	NA	Yes
Ichikawa 2016	Yes	Yes	No	Yes	No	No	Yes	No	Yes	No	Yes	No	NA	No
Inoue 2015	Yes	Yes	Yes	Yes	Yes	No	Yes	No	No	No	Yes	No	NA	No
Kamiya 2016	Yes	Yes	Yes	Yes	No	No	Yes	Yes	No	No	Yes	No	NA	Yes
Kanno 1988	Yes	Yes	NR	Yes	No	No	Yes	NR	CD	CD	Yes	NA	NA	No
Kawakatsu 2015	Yes	Yes	Yes	Yes	Yes	Yes	Yes	Yes	Yes	No	Yes	CD	NA	Yes

(continued)

Author	1	2	3	4	5	6	7	8	9	10	11	12	13	14
Kimura 2010	Yes	Yes	CD	CD	No	No	Yes	No	No	Yes	Yes	No	CD	No
McElligott 2010	Yes	Yes	CD	Yes	Yes	Yes	Yes	Yes	Yes	No	Yes	NR	NA	Yes
McMaster 1996	Yes	Yes	CD	No	No	Yes	No	No	Yes	No	Yes	NR	NA	No
Mudany 2015	Yes	Yes	CD	Yes	NA	Yes	Yes	NA	Yes	Yes	Yes	CD	CD	No
Mukanga 2006	Yes	Yes	Yes	Yes	Yes	Yes	Yes	Yes	Yes	No	Yes	NR	NA	Yes
Nakazawa 2007	Yes	Yes	Yes	Yes	No	No	NA	No	No	Yes	Yes	No	Yes	No
Nokubo 2006	Yes	Yes	CD	Yes	No	No	CD	NR	Yes	No	Yes	No	NA	No
Ogasawara 2016	Yes	Yes	Yes	Yes	No	No	Yes	No	No	No	Yes	No	NA	No
Oguchi 2014	Yes	Yes	Yes	Yes	No	No	Yes	No	Yes	No	Yes	No	NA	No
Osaki 2013	Yes	Yes	CD	Yes	No	Yes	Yes	Yes	Yes	No	Yes	NR	NA	Yes
Shibahara 2010	Yes	Yes	Yes	Yes	No	No	Yes	No	Yes	No	Yes	No	NA	No
Shimizu 2007	Yes	CD	CD	Yes	No	No	Yes	No	No	No	Yes	NA	NR	NA
Tom 2014	Yes	Yes	Yes	Yes	NA	Yes	Yes	Yes	Yes	No	Yes	NR	NA	Yes
Yamagiwa 2009	Yes	Yes	Yes	Yes	No	No	Yes	No	Yes	No	Yes	NA	NA	No
Yamamoto 1998	Yes	Yes	Yes	Yes	No	No	No	No	Yes	No	Yes	NA	NA	No
Yokoi 2019	Yes	Yes	NR	No	No	No	Yes	No	Yes	No	Yes	NA	NA	No
Yuge 2010	Yes	Yes	No	Yes	No	No	Yes	Yes	Yes	No	Yes	NR	NA	No
Zhou 2015	Yes	Yes	Yes	Yes	NA	Yes	Yes	Yes	Yes	Yes	Yes	NR	NR	Yes

1: Was the research question or objective in this paper clearly stated? **2:** Was the study population clearly specified and defined? **3:** Was the participation rate of eligible persons at least 50%? **4:** Were all the subjects selected or recruited from the same or similar populations (including the same time period)? Were inclusion and exclusion criteria for being in the study prespecified and applied uniformly to all participants? **5:** Was a sample size justification, power description, or variance and effect estimates provided? **6:** For the analyses in this paper, were the exposure(s) of interest measured prior to the outcome(s) being measured? **7:** Was the timeframe sufficient so that one could reasonably expect to see an association between exposure and outcome if it existed? **8:** For exposures that can vary in amount or level, did the study examine different levels of the exposure as related to the outcome (e.g., categories of exposure, or exposure measured as continuous variable)? **9:** Were the exposure measures (independent variables) clearly defined, valid, reliable, and implemented consistently across all study participants? **10:** Was the exposure(s) assessed more than once over time? **11:** Were the outcome measures (dependent variables) clearly defined, valid, reliable, and implemented consistently across all study participants? **12:** Were the outcome assessors blinded to the exposure status of participants? **13:** Was loss to follow-up after baseline 20% or less? **14:** Were key potential confounding variables measured and adjusted statistically for their impact on the relationship between exposure(s) and outcome(s)?

Risk of bias assessment of qualitative studies
(Please indicate whether yes, no, or can't tell)

Author	1	2	3	4	5	6	7	8	9	10
Fujii 2020	Yes	Yes	Yes	Can't tell	Yes	Can't tell	Yes	No	Yes	Yes
Yahata 2005	Yes	No	No	No	No	No	Yes	No	Yes	Yes

1: Was there a clear statement of the aims of the research? **2:** Is a qualitative methodology appropriate? **3:** Was the research design appropriate to address the aims of the research? **4:** Was the recruitment strategy appropriate to the aims of the research? **5:** Was the data collected in a way that addressed the research issue? **6:** Has the relationship between researcher and participants been adequately considered? **7:** Have ethical issues been taken into consideration? **8:** Was the data analysis sufficiently rigorous? **9:** Is there a clear statement of findings? **10:** How valuable is the research?

Risk of bias assessment of mixed methods studies
(Please indicate whether yes, no, or can't tell)

Author	1	2	3	4	5	6	7	8	9	10	11	12	13	14	15
Abbott 2013	Yes	Yes	Yes	Yes	Yes	Yes	Yes	Yes	Yes	Yes	Yes	Yes	Yes	No	Yes
Aiga 2016	Yes	Yes	Yes	Yes	Yes	Yes	Yes	Yes	Yes	Yes	Yes	Yes	Yes	No	Yes
Bhuiyan 2006	Yes	Yes	No	No	No	Yes	Yes	Yes	Yes	Yes	Yes	Yes	Yes	No	Yes
Brown 2018	Yes	No	No	No	No	Yes	Yes	Yes	Yes	No	Yes	Yes	Yes	Yes	Yes
Hamilton 2012	Yes	Yes	Yes	Yes	Yes	Yes	No	Yes	Yes	Yes	Yes	Yes	Yes	Yes	Yes

1: Is there an adequate rationale for using a mixed methods design to address the research question? **2:** Are the different components of the study effectively integrated to answer the research question? **3:** Are the outputs of the integration of qualitative and quantitative components adequately interpreted? **4:** Are divergences and inconsistencies between quantitative and qualitative results adequately addressed? **5:** Do the different components of the study adhere to the quality criteria of each tradition of the methods involved? **6:** Is the qualitative approach appropriate to answer the research question? **7:** Are the qualitative data collection methods adequate to address the research question? **8:** Are the findings adequately derived from the data? **9:** Is the interpretation of results sufficiently substantiated by data? **10:** Is there coherence between qualitative data sources, collection, analysis and interpretation? Questions 11-15 depends on whether it involves RCT, non-randomized, or quantitative descriptive studies.
