# Supplementary material for: Roles of the Maternal and Child Health Handbook and Other Home-Based Records on Newborn and Child Health: A Systematic Review
Source: Int J Environ Res Public Health. 2021 Jul 13;18(14):7463. doi: 10.3390/ijerph18147463 (PMC8306696; doi:10.3390/ijerph18147463)
Supplement: Supplementary file 1 [file ijerph-18-07463-s001.zip › Table S7. GRADE table.pdf]

Table S7. GRADE table (newborn)

Question: Should home-based records (intervention) compared to no use of any home-based records (control) be used for improving newborn care seeking?

Quality assessment							№ of participants		Effect		Certainty	Importance
№ of studies	Study design	Risk of bias	Inconsistency	Indirectness	Imprecision	Other considerations	Intervention	Control	Relative (95% CI)	Absolute (95% CI)		
**Care-seeking for newborn complications (Study: Osaki, 2018 (Indonesia))**												
1	randomised trials	serious [a]	not serious	not serious	serious [b]	none	19/183 (10.4%)	9/271 (3.3%)	**OR 3.37** (1.49 to 7.63)	**71 more per 1,000** (from 15 more to 174 more)	⨁⨁◯◯ LOW	CRITICAL
**Care-seeking after delivery (study: Kaneko, 2017 (Burundi))**												
1	observational studies	not serious	not serious	not serious	not serious	none	133/370 (35.9%)	221/344 (64.2%)	**OR 0.31** (0.23 to 0.42)	**285 fewer per 1,000** (from 350 fewer to 212 fewer)	⨁⨁◯◯ LOW	CRITICAL
**Use of laboratory services (study: Haeri Mazanderani (South Africa))**												
1	observational studies	serious [c]	not serious	not serious	not serious	none	326/635 (51.3%)	Not reported	Not calculated	Not calculated	⨁◯◯◯ VERY LOW	IMPORTANT

CI: Confidence interval; **OR:** Odds ratio

Explanations

a. Bias arising from the randomization process and measurement of the outcome
b. Low number of events (< 50)
c. The participation rate of eligible persons and loss to follow-up after baseline cannot be determined.

Question: Should home-based records (intervention) compared to no use of any home-based records (control) be used for improving newborn care knowledge?

Quality assessment							№ of participants		Effect		Certainty	Importance
№ of studies	Study design	Risk of bias	Inconsistency	Indirectness	Imprecision	Other considerations	Intervention	Control	Relative (95% CI)	Absolute (95% CI)		
Immediate breastfeeding (study: Nasir, 2017 (Indonesia))												
1	observational studies	not serious	not serious	not serious	not serious	none	199/214 (93.0%)	176/213 (82.6%)	**OR 2.79** (1.48 to 5.25)	**104 more per 1,000** (from 49 more to 135 more)	⨁⨁◯◯ LOW	CRITICAL
Giving colostrum (study: Nasir, 2017 (Indonesia))												
1	observational studies	not serious	not serious	not serious	not serious	none	199/214 (93.0%)	184/213 (86.4%)	**OR 2.09** (1.09 to 4.02)	**66 more per 1,000** (from 10 more to 98 more)	⨁⨁◯◯ LOW	CRITICAL

Quality assessment							№ of participants		Effect		Certainty	Importance
№ of studies	Study design	Risk of bias	Inconsistency	Indirectness	Imprecision	Other considerations	Intervention	Control	Relative (95% CI)	Absolute (95% CI)		
Exclusive breastfeeding duration (study: Nasir, 2017 (Indonesia))												
1	observational studies	not serious	not serious	not serious	not serious	none	194/214 (90.7%)	156/213 (73.2%)	**OR 3.54** (2.04 to 6.15)	**174 more per 1,000** (from 116 more to 212 more)	⊕⊕◯◯ LOW	CRITICAL
Use of antibiotic for eyes (study: Nasir, 2017 (Indonesia))												
1	observational studies	not serious	not serious	not serious	not serious	none	147/214 (68.7%)	69/213 (32.4%)	**OR 4.58** (3.05 to 6.88)	**363 more per 1,000** (from 270 more to 443 more)	⊕⊕◯◯ LOW	IMPORTANT
Hepatitis B immunization at birth (study: Nasir, 2017 (Indonesia))												
1	observational studies	not serious	not serious	not serious	not serious	none	120/214 (56.1%)	96/213 (45.1%)	**OR 1.56** (1.06 to 2.28)	**111 more per 1,000** (from 14 more to 201 more)	⊕⊕◯◯ LOW	CRITICAL
Thermal protection (study: Nasir, 2017 (Indonesia))												
1	observational studies	not serious	not serious	not serious	not serious	none	208/214 (97.2%)	191/213 (89.7%)	**OR 3.99** (1.59 to 10.06)	**75 more per 1,000** (from 36 more to 92 more)	⊕⊕◯◯ LOW	IMPORTANT
Cord care (study: Nasir, 2017 (Indonesia))												
1	observational studies	not serious	not serious	not serious	not serious	none	171/214 (79.9%)	66/213 (31.0%)	**OR 8.86** (5.69 to 13.79)	**489 more per 1,000** (from 409 more to 551 more)	⊕⊕◯◯ LOW	IMPORTANT
Recognize the danger signs in newborns (study: Nasir, 2017 (Indonesia))												
1	observational studies	not serious	not serious	not serious	not serious	none	170/214 (79.4%)	115/213 (54.0%)	**OR 3.29** (2.15 to 5.05)	**254 more per 1,000** (from 176 more to 316 more)	⊕⊕◯◯ LOW	CRITICAL

CI: Confidence interval; **OR:** Odds ratio

Question: Should home-based records (intervention) compared to no use of any home-based records (control) be used for improving newborn care practices?

Certainty assessment							№ of participants		Effect		Certainty	Importance
№ of studies	Study design	Risk of bias	Inconsistency	Indirectness	Imprecision	Other considerations	Intervention	Control	Relative (95% CI)	Absolute (95% CI)		
**Immediate breastfeeding (study: Mori, 2015 (Mongolia))**												
1	randomised trials	serious [a]	not serious	not serious	not serious	12 participants in the control group received the intervention	251/252 (99.6%)	244/246 (99.2%)	**RR 1.07** (0.97 to 1.18)	**69 more per 1,000** (from 30 fewer to 179 more)	⨁⨁⨁◯ MODERATE	CRITICAL
**Good newborn care and self-care (studies: Shah, 1993 (multi-countries); Nasir, 2017 (Indonesia))**												
2	observational studies	serious [b]	not serious	not serious	serious [c]	none	Not reported	Not reported	**OR 1.81** (1.24 to 2.66)	**2 fewer per 1,000** (from 3 fewer to 1 fewer)	⨁◯◯◯ VERY LOW	CRITICAL

CI: Confidence interval; **RR:** Risk ratio; **OR:** Odds ratio

Explanations

a. Bias in measurement of the outcome.
b. Serious concerns regarding confounding variables and selective outcome reporting in Shah, 1993
c. Number of events not reported.

Question: Should home-based records (intervention) compared to no use of any home-based records (control) be used for improving perinatal mortality and morbidity?

Qualityassessment							№ of participants		Effect		Certainty	Importance
№ of studies	Study design	Risk of bias	Inconsistency	Indirectness	Imprecision	Other considerations	Intervention	Control	Relative (95% CI)	Absolute (95% CI)		
**Neonatal deaths (study: Mori, 2015 (Mongolia))**												
1	randomised trials	serious [a]	not serious	not serious	very serious [b]	12 participants in the control group received the intervention	1/253 (0.4%)	2/248 (0.8%)	**RR 1.00** (0.99 to 1.02)	**0 fewer per 1,000** (from 0 fewer to 0 fewer)	⨁◯◯◯ VERY LOW	IMPORTANT
**APGAR score (study: Mori, 2015 (Mongolia))**												
1	randomised trials	serious [a]	not serious	not serious	not serious	12 participants in the control group received the intervention	Mean (SD): 7.55 (0.89)	Mean (SD): 7.34 (1.25)	Mean difference: 0.21 (-0.21-0.63)	Not calculated	⨁⨁⨁◯ MODERATE	IMPORTANT

CI: Confidence interval; **RR:** Risk ratio

Explanations

a. Bias in measurement of the outcome.
b. Very low number of events (<100)

Question: Should a different type of home-based record (intervention) compared to a standard home-based record (control) be used for improving newborn care knowledge?

Quality assessment							№ of participants		Effect		Certainty	Importance
№ of studies	Study design	Risk of bias	Inconsistency	Indirectness	Imprecision	Other considerations	Intervention	Control	Relative (95% CI)	Absolute (95% CI)		
**Immediate breastfeeding (assessed with: Yanagisawa, 2015 (Cambodia))**												
1	randomised trials	serious [a]	not serious	not serious	serious [b]	none	Pre-intervention: 23.8% Post-intervention: 40.0% Difference: 16.2%	Pre-intervention: 30.0% Post-intervention: 40.0% Difference: 10.0%	Difference-in-differences: 6.2% OR not reported	Not calculated	⨁⨁◯◯ LOW	CRITICAL

CI: Confidence interval

Explanations

a. Bias arising from the randomization process.
b. The sample size and event numbers are not available.

Question: Should a different type of home-based record (intervention) compared to a standard home-based record (control) be used for improving newborn care practices?

Quality assessment							№ of participants		Effect		Certainty	Importance
№ of studies	Study design	Risk of bias	Inconsistency	Indirectness	Imprecision	Other considerations	Intervention	Control	Relative (95% CI)	Absolute (95% CI)		
**Immediate breastfeeding (assessed with: Lovell, 1987 (UK))**												
1	randomised trials	serious [a]	not serious	not serious	serious [b]	none	77/98 (78.6%)	81/105 (77.1%)	**OR 1.09** (0.56 to 2.11)	**15 more per 1,000** (from 117 fewer to 105 more)	⨁⨁◯◯ LOW	CRITICAL

CI: Confidence interval; **OR:** Odds ratio

Explanations

a. Bias due to deviations from intended intervention and in the measurement of the outcome
b. The population comprised of a higher proportion of one-parent families, high unemployment rate, and a quarter sample included west Indian and other racial groups disproportionately affected by social deprivation.

Question: Should a different type of home-based record (intervention) compared to a standard home-based record (control) be used for improving perinatal mortality and morbidity
?

Quality assessment							№ of participants		Effect		Certainty	Importance
№ of studies	Study design	Risk of bias	Inconsistency	Indirectness	Imprecision	Other considerations	Intervention	Control	Relative (95% CI)	Absolute (95% CI)		
**Neonatal death or stillborn (assessed with: Lovell, 1987 (UK))**												
1	randomised trials	serious [a]	not serious	serious [b]	very serious [c]	none	2/104 (1.9%)	2/104 (1.9%)	**OR 1.04** (0.14 to 7.52)	**1 more per 1,000** (from 16 fewer to 109 more)	⨁◯◯◯ VERY LOW	CRITICAL

CI: Confidence interval; **OR:** Odds ratio

Explanations

a. Bias due to deviations from intended intervention and in the measurement of the outcome
b. The population comprised of a higher proportion of one-parent families, high unemployment rate, and a quarter sample included west Indian and other racial groups disproportionately affected by social deprivation
c. Wide confidence interval and small number of events (<10)

Table S7. GRADE Table (children)

Question: Should home-based records (intervention) compared to no use of any home-based records (control) be used for improving children vaccination use/uptake?

Quality assessment							№ of participants		Effect		Certainty	Importance
№ of studies	Study design	Risk of bias	Inconsistency	Indirectness	Imprecision	Other considerations	Intervention	Control	Relative (95% CI)	Absolute (95% CI)		
**DTP3 completion (study: Lakhani, 1984 (UK); Stille, 2001 (USA))**												
2	randomised trials	very serious [a]	not serious	serious [b]	not serious	none	126/313 (40.3%)	136/301 (45.2%)	**RR 0.89** (0.64 to 1.24)	**50 fewer per 1,000** (from 163 fewer to 108 more)	⨁◯◯◯ VERY LOW	CRITICAL
**Rotavirus vaccination (study: Inoue, 2015 (Japan))**												
1	observational studies	very serious [c]	not serious	not serious	not serious	none	126/376 (33.5%)	No control group	Not reported	Not calculated	⨁◯◯◯ VERY LOW	CRITICAL
**BCG and polio vaccination (study: Yamamoto, 1998 (Japan))**												
1	observational studies	very serious [d]	not serious	not serious	not serious	none	281/302 (93.0%)	No control group	Not reported	Not calculated	⨁◯◯◯ VERY LOW	CRITICAL
**Mumps, measles, chicken pox (study: Kimura, 2010 (Japan))**												
1	observational studies	very serious [c]	not serious	not serious	serious [e]	none	Number of events not reported	No control group	Vaccination coverage: Before elementary: 8.5-13.9% 6th graders: 8.5-13.2%	Not calculated	⨁◯◯◯ VERY LOW	CRITICAL

CI: Confidence interval; **RR:** Risk ratio

Explanations

a. Bias in measurement of the outcome and Stille (2001) was a non-randomized design.
b. Differences in intervention design and DTP3 completion measurement
c. Exposure measures are not clearly defined, and key potential confounding variables not measured and adjusted statistically.
d. Some methodological considerations and key potential confounding variables not measured and adjusted statistically.
e. Unable to assess the number of events as not reported

Question: Should home-based records (intervention) compared to no use of any home-based records (control) be used for improving vaccination history/records?

Quality assessment							№ of participants		Effect		Certainty	Importance
№ of studies	Study design	Risk of bias	Inconsistency	Indirectness	Imprecision	Other considerations	home-based records	no use of any home-based records	Relative (95% CI)	Absolute (95% CI)		
**Vaccination history/records (studies: Kanno, 1988 (Japan); Jeffs, 1994 (Australia); McMaster, 1996 (Bosnia and Herzegovina); Kreuter, 2004 (USA); Mukanga, 2006 (Uganda); Shimizu, 2007 (Dominican Republic); McElligott, 2010 (USA); Abbott, 2013 (Australia); Ogasawara, 2016 (Japan))**												
9	observational studies	serious [a]	serious [b]	serious [c]	not serious	none	Not estimable	Not estimable	More up-to-date immunization status seen in the intervention group	Not calculated	⨁◯◯◯ VERY LOW	CRITICAL

CI: Confidence interval

Explanations

a. Some methodological considerations and key potential confounding variables not measured nor adjusted statistically.
b. McMaster (1996) reported a mixed effect on the outcome; all others reported a significant effect.
c. Differences in the measurement of the outcome.

Question: Should home-based records (intervention) compared to no use of any home-based records (control) be used for improving childcare-seeking?

Quality assessment							№ of participants		Effect		Certainty	Importance
№ of studies	Study design	Risk of bias	Inconsistency	Indirectness	Imprecision	Other considerations	Intervention	Control	Relative (95% CI)	Absolute (95% CI)		
**Care-seeking for child illnesses (RCT) (studies: Osaki, 2018 (Indonesia); Grøvdal, 2006 (Norway))**												
2	randomised trials	serious [a]	not serious	serious [b]	very serious [c]	none	Osaki (2018): 75.8% sought care among the reported child illnesses	Osaki (2018): 71.2% sought care among the reported child illnesses Grøvdal (2006): 17% more parents in the control group sought care for children with chronic diseases	Not reported	Not calculated	⨁◯◯◯ VERY LOW	CRITICAL
**Care-seeking for child illnesses (observational study) (studies: Kawakatsu, 2015 (Kenya); Zhou, 2015 (USA))**												
2	observational studies	not serious	not serious	not serious	serious [d]	none	Kawakatsu (2015): Fever: 1012/1331 (76.0%) Diarrhea: 566/1331 (42.5%)<br><br>Zhou (2015): Unable to assess the number of events as not reported	Kawakatsu (2015): Fever: 476/652 (73.0%) Diarrhea: 266/652 (40.8%)<br><br>Zhou (2015): Unable to assess the number of events as not reported	Kawakatsu (2015): Impact of 9.4 and 12.6 percentage points for care-seeking for fever and diarrhea, respectively Zhou (2015): 21% more outpatient clinic visits and 26% more telephone encounters	Not calculated	⨁◯◯◯ VERY LOW	CRITICAL

Quality assessment							№ of participants		Effect		Certainty	Importance
№ of studies	Study design	Risk of bias	Inconsistency	Indirectness	Imprecision	Other considerations	Intervention	Control	Relative (95% CI)	Absolute (95% CI)		
**Use of health care and laboratory services (RCT) (study: Grøvdal, 2006 (Norway))**												
1	randomised trials	serious [a]	not serious	not serious	very serious [e]	none	Children with more encounters with healthcare services Non-routine child health center (CHC): 35/155 Doctor outside CHC: 30/155 Specialist or hospital: 13/155	Children with more encounters with healthcare services Non-routine child health center (CHC): 35/154 Doctor outside CHC: 28/154 Specialist or hospital: 16/154	Non-routine child health center (CHC): OR 0.99 (0.58-1.69) Doctor outside CHC: OR 1.08 (0.61-1.91) Specialist or hospital: OR 0.79 (0.37-1.70)	Not calculated	⊕◯◯◯ VERY LOW	CRITICAL
**Use of health care and laboratory services (observational study) (studies: Nakazawa, 2007 (Japan); Mudany, 2015 (Kenya))**												
2	observational studies	serious [f]	not serious	serious [g]	not serious	none	Nakazawa (2007): 21/35 had normal hearing<br><br>Mudany (2015): HIV DNA testing in infants rose from 27,000 (2007) to 55,000 (2010) to 60,000 (2012)	No control group	Not reported	Not calculated	⊕◯◯◯ VERY LOW	CRITICAL
**Adherence to recommended immunizations (study: Tom, 2014 (USA))**												
1	observational studies	not serious	not serious	not serious	not serious	none	KP Hawaii: 766 KP Northwest: 2795	KP Hawaii: 766 KP Northwest: 2795	KP Northwest: OR 1.2 (1.0-1.3)	Not calculated	⊕⊕◯◯ LOW	CRITICAL
**Adherence to childcare visit recommendations (study: Tom, 2014 (USA))**												
1	observational studies	not serious	not serious	not serious	not serious	none	KP Hawaii: 766 KP Northwest: 2795	KP Hawaii: 766 KP Northwest: 2795	KP Hawaii: OR 1.9 (1.3-2.9)<br><br>KP Northwest: OR 2.5 (2.1-2.9)	Not calculated	⊕⊕◯◯ LOW	CRITICAL

CI: Confidence interval

Explanations

a. Bias in measurement of the outcome.
b. Population is children with chronic diseases in Grøvdal (2006)
c. Unable to assess the number of events as not reported in Grøvdal (2006)
d. Unable to assess the number of events as not reported
e. Unable to assess the number of events as outcome data are ordinal
f. Some methodological considerations and key potential confounding variables not measured nor adjusted statistically.
g. Differences in outcome measurement.

Question: Should home-based records (intervention) compared to no use of any home-based records (control) be used for improving child healthcare knowledge?

Quality assessment							№ of participants		Effect		Certainty	Importance
№ of studies	Study design	Risk of bias	Inconsistency	Indirectness	Imprecision	Other considerations	Intervention	Control	Relative (95% CI)	Absolute (95% CI)		
**General health (RCT) (assessed with: Grøvdal, 2006 (Norway))**												
1	randomised trials	serious [a]	not serious	not serious	very serious [b]	none	Parents with improved knowledge on child's health Newborns get the best view of me at a distance of 60 cm: 21/155 Call a doctor when child's temperature passes 39°C: 44/155 Let the child use a baby-walker or jumping-reins: 10/155	Parents with improved knowledge on child's health Newborns get the best view of me at a distance of 60 cm: 23/154 Call a doctor when child's temperature passes 39°C: 44/154 Let the child use a baby-walker or jumping-reins: 17/154	Newborns get the best view of me at a distance of 60 cm: OR 0.89 (0.47-1.69) Call a doctor when child's temperature passes 39°C: OR 0.99 (0.60-1.62) Let the child use a baby-walker or jumping-reins: OR 0.56 (0.25-1.26)	Not calculated	⨁◯◯◯ VERY LOW	CRITICAL
**General health (observational study) (assessed with: Moore, 2000 (UK); Kawakatsu, 2015 (Kenya))**												
2	observational studies	very serious [c]	serious [d]	not serious	serious [e]	none	Moore (2000): Not reported Kawakatsu (2015): Higher health knowledge: 1331/1331 (100%)	Moore (2000): Not reported Kawakatsu (2015): Higher health knowledge: 650/652 (99.7%)	Moore (2000): Not reported Kawakatsu (2015): Impact of 5.9 percentage points for higher health knowledge was statistically significant among users in the intervention group	Not calculated	⨁◯◯◯ VERY LOW	CRITICAL
**Immunization (assessed with: Oguchi, 2014 (Japan); Kamiya, 2016 (Japan); Nasir, 2017 (Indonesia))**												
3	observational studies	very serious [f]	not serious	not serious	not serious	none	Oguchi (2014): Motivation to vaccinate among mothers: 365/523 (70%) Kamiya (2016): Requested information on the vaccination schedule: 69/123 (56.1%)	No control group	Oguchi (2014): 70% of mothers was motivated to vaccinate their child Kamiya (2016): 56.1% of mothers requested information on the vaccination schedule	Not calculated	⨁◯◯◯ VERY LOW	CRITICAL
**Exclusive breastfeeding (assessed with: Hagiwara, 2013 (Palestine); Aiga, 2016 (Vietnam))**												

Quality assessment							№ of participants		Effect		Certainty	Importance
№ of studies	Study design	Risk of bias	Inconsistency	Indirectness	Imprecision	Other considerations	Intervention	Control	Relative (95% CI)	Absolute (95% CI)		
2	observational studies	not serious	not serious	not serious	not serious	none	Hagiwara (2013): Number of events not reported<br>Aiga (2016): Pre-intervention: 529/800 (66.1%)<br>Post-intervention: 702/810 (86.7%)	No control group	Hagiwara (2013): Significant improvement on knowledge about exclusive breastfeeding among less educated women who are literate (p<0.05)<br>Aiga (2016): 20.6% of pregnant women and mothers showed improved knowledge on exclusive breastfeeding	Not calculated	⊕⊕◯◯<br>LOW	CRITICAL
**Detection of biliary atresia (assessed with: Yamagiwa, 2009 (Japan); Hirayama, 2011 (Japan); Yokoi, 2019 (Japan))**												
3	observational studies	serious [f]	serious [g]	not serious	not serious	none	Yamagiwa (2009): Knowledge on biliary atresia: 27/59<br>Know the stool color of biliary atresia: 14/27<br>Interest in biliary atresia: 52/59<br><br>Hirayama (2011)<br>Heard of biliary atresia: 77/239 (32.2%)<br>Interest in biliary atresia: 137/237 (57.8%)<br>Using the stool color card: 203/239 (84.9%)<br><br>Yokoi (2019): Usefulness of stool color card: 36/41 (87.8%)	Yamagiwa (2009): Knowledge on biliary atresia: 19/58<br>Know the stool color of biliary atresia: 3/18<br>Interest in biliary atresia: 39/57	Yamagiwa (2009): Knowledge on biliary atresia: OR 1.73 (0.82-3.67)<br>Know the stool color of biliary atresia: OR 5.38 (1.26-22.99)<br>Interest in biliary atresia: OR 3.43 (1.30-9.01)<br><br>Hirayama (2011) and Yokoi (2019): not estimable	Not calculated	⊕◯◯◯<br>VERY LOW	CRITICAL
**Sudden infant syndrome (assessed with: Ichikawa, 2016 (Japan))**												
1	observational studies	serious [f]	not serious	not serious	not serious	none	Knowledge with sudden infant death syndrome (SIDS): 118/378 (31.2%)<br>Heard about SIDS: 234/378 (61.9%)	No control group	31.2% of mothers were knowledgeable about SIDS while 61.9% have heard it	Not calculated	⊕◯◯◯<br>VERY LOW	CRITICAL
**Accident prevention of infant (assessed with: Nokubo, 2006 (Japan))**												
1	observational studies	serious [f]	not serious	not serious	not serious	none	For those who have read the MCH handbook: Practice accident prevention: 49/55 (89.1%)	For those who did not read the MCH handbook: Practice accident prevention: 8/14(57.1%)	Accident prevention of infant: OR 6.13 (1.58-23.77)		⊕◯◯◯<br>VERY LOW	CRITICAL

CI: Confidence interval

Explanations

a. Bias in measurement of the outcome
b. Unable to assess the number of events as outcome data are ordinal
c. Bias in measurement of the outcome and selection of the reported result in Moore (2000)
d. Moore (2000) reports no effect on outcome; Kawakatsu (2015) reports a significant effect
e. No data was presented in Moore (2000)
f. Some methodological concerns and key potential confounding variables not measured nor adjusted statistically
g. Yamagiwa (2009) and Yokoi (2019) reported a mixed effect on outcome; Hirayama (2011) reports a significant effect

Question: Should home-based records (intervention) compared to no use of any home-based records (control) be used for improving child healthcare practices?

Quality assessment							№ of participants		Effect		Certainty	Importance
№ of studies	Study design	Risk of bias	Inconsistency	Indirectness	Imprecision	Other considerations	Intervention	Control	Relative (95% CI)	Absolute (95% CI)		
**Exclusive breastfeeding (assessed with: Osaki, 2018 (Indonesia))**												
1	randomised trials	serious [a]	not serious	not serious	serious [b]	none	79/183 (43.2%)	132/271 (48.7%)	**OR 0.76** (0.51 to 1.14)	**68 fewer per 1,000** (from 161 fewer to 33 more)	⨁⨁◯◯ LOW	CRITICAL
**Exclusive breastfeeding (assessed with: Aiga, 2016 (Vietnam))**												
1	observational studies	not serious	not serious	not serious	not serious	none	Pre-intervention: 146/800 (18.3%)<br>Post-intervention: 607/810 (74.9%)	No control group	Not reported	Not calculated	⨁⨁◯◯ LOW	CRITICAL
**Continued breastfeeding (assessed with: Osaki, 2018 (Indonesia))**												
1	randomised trials	serious [a]	not serious	not serious	not serious	none	167/183 (91.3%)	224/271 (82.7%)	**OR 2.31** (1.22 to 4.39)	**90 more per 1,000** (from 27 more to 128 more)	⨁⨁⨁◯ MODERATE	CRITICAL
**Complementary Feeding (assessed with: Osaki, 2018 (Indonesia))**												
1	randomised trials	serious [a]	not serious	not serious	not serious	none	113/183 (61.7%)	74/271 (27.3%)	**OR 4.35** (2.85 to 6.65)	**347 more per 1,000** (from 244 more to 441 more)	⨁⨁⨁◯ MODERATE	CRITICAL
**Proper feeding order (assessed with: Osaki, 2018 (Indonesia))**												
1	randomised trials	serious [a]	not serious	not serious	not serious	none	93/183 (50.8%)	73/271 (26.9%)	**OR 2.70** (1.79 to 4.09)	**229 more per 1,000** (from 128 more to 332 more)	⨁⨁⨁◯ MODERATE	CRITICAL

Quality assessment							№ of participants		Effect		Certainty	Importance
№ of studies	Study design	Risk of bias	Inconsistency	Indirectness	Imprecision	Other considerations	Intervention	Control	Relative (95% CI)	Absolute (95% CI)		
**Varied foods feeding (assessed with: Osaki, 2018 (Indonesia))**												
1	randomised trials	serious [a]	not serious	not serious	not serious	none	Fruits and/or fruits extract: 71/183 (38.8%)<br>Add Protein/ vitamin/ oil rich food to soft rice: 89/183 (48.6%)<br>Various snack food for two times between meals: 107/183 (58.5%)	Fruits and/or fruits extract: 62/271 (22.9%)<br>Add Protein/ vitamin/ oil rich food to soft rice: 101/271 (37.3%)<br>Various snack food for two times between meals: 70/271 (25.8%)	Fruits and/or fruits extract: **OR 2.18** (1.42 to 3.36)<br>Add Protein/ vitamin/ oil rich food to soft rice: **OR 1.54** (1.03 to 2.30)<br>Various snack food for two times between meals: **OR 4.14** (2.70 to 6.34)	Not calculated	⊕⊕⊕◯<br>MODERATE	CRITICAL
**Self-feeding training (assessed with: Osaki, 2018 (Indonesia))**												
1	randomised trials	serious [a]	not serious	not serious	not serious	none	65/183 (35.5%)	45/271 (16.6%)	**OR 2.75** (1.74 to 4.36)	**188 more per 1,000** (from 91 more to 299 more)	⊕⊕⊕◯<br>MODERATE	IMPORTANT
**Recording immunizations (assessed with: Lakhani, 1984 (UK))**												
1	randomised trials	very serious [c]	not serious	not serious	not serious	none	The proportion of booklets with entries on this page is low.	The information was still being recorded on an immunization card that had been used previously.	Not reported	Not calculated	⊕⊕◯◯<br>LOW	CRITICAL
**Recording immunizations (assessed with: Mukanga, 2006 (Uganda); Brown, 2018 (Kenya); Enokido, 1965 (Japan); Fujimoto, 2001 (Japan))**												
4	observational studies	serious [d]	serious [e]	not serious	not serious	none	Mukanga (2006): Card seen, record immunization: 160/172 (93.0%) Card not seen, record immunization: 81/88 (92.0%)<br><br>Brown (2018): Vaccination history (82%)<br><br>Enokido (1965): Vaccination records (30.7%)<br><br>Fujimoto (2001): Most useful page was the vaccination record		Mukanga (2006): **OR 1.63** (0.44 to 3.17)	Not calculated	⊕◯◯◯<br>VERY LOW	CRITICAL
**Growth monitoring (assessed with: Mukanga, 2006 (Uganda); Hamilton, 2012 (Australia); Araujo, 2017 (Brazil); Enokido, 1965 (Japan); Hokama, 2000 (Japan); Aoki, 2009 (Japan); Shibahara, 2010 (Japan); Fujii, 2020 (Japan))**												
8	observational studies	serious [d]	serious [e]	not serious	not serious	none	Mixed results were obtained for growth monitoring.		Not calculated	Not calculated	⊕◯◯◯<br>VERY LOW	CRITICAL

CI: Confidence interval; **OR:** Odds ratio

Explanations

a. Bias in measurement of the outcome and some concerns in the randomization process.
b. Low number of events (<300)
c. Bias due to deviations from intended intervention, missing outcome data, measurement of the outcome.
d. Some methodological considerations and key potential confounding variables not measured nor adjusted statistically.
e. Findings showed mixed results.

Question: Should home-based records (intervention) compared to no use of any home-based records (control) be used for infant and child illness management?

Quality assessment							№ of participants		Effect		Certainty	Importance
№ of studies	Study design	Risk of bias	Inconsistency	Indirectness	Imprecision	Other considerations	Intervention	Control	Relative (95% CI)	Absolute (95% CI)		
**Home care for cough (assessed with: Osaki, 2018 (Indonesia))**												
1	randomised trials	serious [a]	not serious	not serious	serious [b]	none	36/45 (80.0%)	32/60 (53.3%)	**OR 3.50** (1.44 to 8.52)	**267 more per 1,000** (from 89 more to 374 more)	⨁⨁◯◯ LOW	CRITICAL
**Home care for diarrhea (assessed with: Osaki, 2018 (Indonesia))**												
1	randomised trials	serious [a]	not serious	serious [c]	serious [b]	none	20/24 (83.3%)	25/27 (92.6%)	**OR 0.40** (0.07 to 2.40)	**93 fewer per 1,000** (from 459 fewer to 42 more)	⨁◯◯◯ VERY LOW	CRITICAL
**Home care for diarrhea (assessed with: Shimizu, 2007 (Dominican Republic))**												
1	observational studies	serious [d]	not serious	very serious [e]	not serious	none	Common problems such as diarrhea decreased. No data was presented.	No control group	Not calculated	Not calculated	⨁◯◯◯ VERY LOW	CRITICAL
**Vitamin A use (assessed with: Osaki, 2018 (Indonesia))**												
1	randomised trials	serious [a]	not serious	not serious	not serious	none	160/183 (87.4%)	205/271 (75.6%)	**OR 2.00** (1.16 to 3.47)	**105 more per 1,000** (from 26 more to 159 more)	⨁⨁⨁◯ MODERATE	CRITICAL

CI: Confidence interval; **OR:** Odds ratio

Explanations

a. Bias in measurement of the outcome and some concerns in the randomization process.
b. Very low number of events (<100)
c. Diarrhea is only one of the possible illnesses.
d. Some methodological considerations and key potential confounding variables not measured nor adjusted statistically.
e. No data was presented.

Question: Should home-based records (intervention) compared to no use of any home-based records (control) be used for improving child mortality and morbidity?

Quality assessment							№ of participants		Effect		Certainty	Importance
№ of studies	Study design	Risk of bias	Inconsistency	Indirectness	Imprecision	Other considerations	Intervention	Control	Relative (95% CI)	Absolute (95% CI)		
**Underweight children (assessed with: Osaki, 2018 (Indonesia))**												
1	randomised trials	serious [a]	not serious	not serious	very serious [b]	none	7/135 (5.2%)	35/250 (14.0%)	**OR 0.33** (0.12 to 0.94)	**89 fewer per 1,000** (from 121 fewer to 7 fewer)	⊕◯◯◯ VERY LOW	IMPORTANT
**Stunted growth (assessed with: Osaki, 2018 (Indonesia))**												
1	randomised trials	serious [a]	not serious	not serious	serious [c]	none	35/133 (26.3%)	100/248 (40.3%)	**OR 0.53** (0.30 to 0.92)	**140 fewer per 1,000** (from 235 fewer to 20 fewer)	⊕⊕◯◯ LOW	IMPORTANT
**Wasting (assessed with: Osaki, 2018 (Indonesia))**												
1	randomised trials	serious [a]	not serious	not serious	very serious [b]	none	10/133 (7.5%)	30/248 (12.1%)	**OR 0.59** (0.24 to 1.47)	**46 fewer per 1,000** (from 89 fewer to 47 more)	⊕◯◯◯ VERY LOW	IMPORTANT
**Risk of cognitive delay (assessed with: Dagvadorj, 2017 (Mongolia))**												
1	randomised trials	serious [d]	not serious	not serious	very serious [b]	none	17/214 (7.9%)	24/172 (14.0%)	**OR 0.32** (0.14 to 0.73)	**90 fewer per 1,000** (from 117 fewer to 34 fewer)	⊕◯◯◯ VERY LOW	IMPORTANT

CI: Confidence interval; **OR:** Odds ratio

Explanations

a. Bias in measurement of the outcome and some concerns in the randomization process.
b. Very low number of events (<100)
c. Low number of events (<300)
d. Bias in measurement of the outcome and selection of the reported result.

Question: Should home-based records (intervention) compared to no use of any home-based records (control) be used for improving the continuum of care?

Quality assessment							№ of participants		Effect		Certainty	Importance
№ of studies	Study design	Risk of bias	Inconsistency	Indirectness	Imprecision	Other considerations	Intervention	Control	Relative (95% CI)	Absolute (95% CI)		
**Continuum of care (Maternal and newborn: TT2, ANC4, SBA,) (assessed with: Osaki, 2018 (Indonesia))**												
1	randomised trials	serious [a]	not serious	not serious	serious [b]	none	53/183 (29.0%)	50/271 (18.5%)	**OR 1.46** (0.89 to 2.40)	**64 more per 1,000** (from 17 fewer to 167 more)	⨁⨁◯◯ LOW	CRITICAL
**Continuum of care (Maternal and newborn: TT2, ANC4, SBA, VitA, ExBF) (assessed with: Osaki, 2018 (Indonesia))**												
1	randomised trials	serious [a]	not serious	not serious	very serious [c]	none	31/183 (16.9%)	22/271 (8.1%)	**OR 2.38** (1.22 to 4.64)	**93 more per 1,000** (from 16 more to 210 more)	⨁◯◯◯ VERY LOW	CRITICAL
**Continuum of care (Maternal and newborn: TT2, ANC4, SBA, VitA, ExBF, started CF in 6-9 months) (assessed with: Osaki, 2018 (Indonesia))**												
1	randomised trials	serious [a]	not serious	not serious	very serious [c]	Large effect in positive direction, but wide CIs	22/183 (12.0%)	5/271 (1.8%)	**OR 7.13** (2.43 to 20.90)	**100 more per 1,000** (from 25 more to 264 more)	⨁◯◯◯ VERY LOW	CRITICAL
**Continuum of care (assessed with: Shah, 1993 (Multi-countries); Aiga, 2016 (Vietnam); Kaneko, 2017 (Burundi); Osaki, 2013 (Indonesia); Shimizu, 2007 (Dominican Republic); Yuge, 2010 (Japan); Adachi, 2010 (Japan))**												
7	observational studies	serious [d]	not serious	not serious	not serious	none	Not estimable	Not estimable	Not calculated	Not calculated	⨁◯◯◯ VERY LOW	CRITICAL

CI: Confidence interval; **OR:** Odds ratio

Explanations

a. Bias in measurement of the outcome and some concerns in the randomization process.
b. A low number of events (<300)
c. A very low number of events (<100) and wide confidence intervals
d. Some methodological considerations and key potential confounding variables not measured nor adjusted statistically.

Question: Should a different type of home-based record (intervention) compared to a standard home-based record (control) be used for improving children vaccination use/uptake?

Quality assessment							№ of participants		Effect		Certainty	Importance
№ of studies	Study design	Risk of bias	Inconsistency	Indirectness	Imprecision	Other considerations	Intervention	Control	Relative (95% CI)	Absolute (95% CI)		
**DTP3 completion (assessed with: Usman, 2009 (Pakistan); Usman, 2011 (Pakistan))**												
2	randomised trials	serious [a]	not serious	not serious	not serious	none	511/753 (67.9%)	354/753 (47.0%)	**OR 2.39** (1.45 to 3.92)	**209 more per 1,000** (from 93 more to 307 more)	⨁⨁⨁◯ MODERATE	CRITICAL

CI: Confidence interval; **OR:** Odds ratio

Explanations

a. Bias in measurement of the outcome.

Question: Should a different type of home-based record (intervention) compared to a standard home-based record (control) be used for improving childcare-seeking?

Quality assessment							№ of participants		Effect		Certainty	Importance
№ of studies	Study design	Risk of bias	Inconsistency	Indirectness	Imprecision	Other considerations	Intervention	Control	Relative (95% CI)	Absolute (95% CI)		
**Adherence to the recommended immunizations (assessed with: Bhuiyan, 2006 (Bangladesh))**												
1	observational studies	serious [a]	not serious	not serious	serious [b]	none	8.3% of the mothers adhered to the recommended vaccination	1.5% of the mothers adhered to the recommended vaccination	Not calculated	Not calculated	⨁◯◯◯ VERY LOW	CRITICAL

CI: Confidence interval

Explanations

a. The integration and inconsistencies between quantitative and qualitative results are not adequately addressed.
b. Unable to assess the number of events as not reported

Question: Should a different type of home-based record (intervention) compared to a standard home-based record (control) be used for improving child healthcare knowledge?

Quality assessment							№ of participants		Effect		Certainty	Importance
№ of studies	Study design	Risk of bias	Inconsistency	Indirectness	Imprecision	Other considerations	Intervention	Control	Relative (95% CI)	Absolute (95% CI)		
**Immunization (assessed with: Bhuiyan, 2006 (Bangladesh))**												
1	observational studies	serious [a]	not serious	not serious	serious [b]	none	32.4% of mothers showed significant improvement in knowledge about immunization	5.7% of mothers showed improvement in knowledge about immunization	Not calculated	Not calculated	⨁◯◯◯ VERY LOW	CRITICAL
**Exclusive breastfeeding (assessed with: Bhuiyan, 2006 (Bangladesh))**												
1	observational studies	serious [a]	not serious	not serious	serious [b]	none	28.7% of mothers showed significant improvement in knowledge about exclusive breastfeeding	4.6% of mothers showed improvement in knowledge about exclusive breastfeeding	Not calculated	Not calculated	⨁◯◯◯ VERY LOW	CRITICAL

CI: Confidence interval

Explanations

a. The integration and inconsistencies between quantitative and qualitative results are not adequately addressed.
b. Unable to assess the number of events as not reported

Question: Should a different type of home-based record (intervention) compared to a standard home-based record (control) be used for improving child healthcare practices?

Quality assessment							№ of participants		Effect		Certainty	Importance
№ of studies	Study design	Risk of bias	Inconsistency	Indirectness	Imprecision	Other considerations	Intervention	Control	Relative (95% CI)	Absolute (95% CI)		
**Exclusive breastfeeding (assessed with: Bhuiyan, 2006 (Bangladesh))**												
1	observational studies	serious [a]	not serious	not serious	serious [b]	none	16.9% of the mothers practiced exclusive breastfeeding	0.7% of the mothers practiced exclusive breastfeeding	Not calculated	Not calculated	⨁◯◯◯ VERY LOW	CRITICAL

CI: Confidence interval

Explanations

a. The integration and inconsistencies between quantitative and qualitative results are not adequately addressed.
b. Unable to assess the number of events as not reported

Question: Should a different type of home-based record (intervention) compared to a standard home-based record (control) be used for improving infant and child illness management?

Quality assessment							№ of participants		Effect		Certainty	Importance
№ of studies	Study design	Risk of bias	Inconsistency	Indirectness	Imprecision	Other considerations	Intervention	Control	Relative (95% CI)	Absolute (95% CI)		
**Vitamin A use (assessed with: Bhuiyan, 2006 (Bangladesh))**												
1	observational studies	serious [a]	not serious	not serious	serious [b]	none	17.6% of the mothers used Vitamin A	1.4% of the mothers used Vitamin A	Not calculated	Not calculated	⨁◯◯◯ VERY LOW	CRITICAL

CI: Confidence interval

Explanations

a. The integration and inconsistencies between quantitative and qualitative results are not adequately addressed.
b. Unable to assess the number of events as not reported
