# Supplementary material for: Roles of the Maternal and Child Health Handbook and Other Home-Based Records on Newborn and Child Health: A Systematic Review
Source: Int J Environ Res Public Health. 2021 Jul 13;18(14):7463. doi: 10.3390/ijerph18147463 (PMC8306696; doi:10.3390/ijerph18147463)
Supplement: Supplementary file 1 [file ijerph-18-07463-s001.zip › Text S1. PROSPERO Registration CRD42020166545.pdf]

Roles of Maternal and Child Health Handbook and other Home-based Records on Newborn and Child Health: A Systematic Review

Rogie Royce Carandang, Jennifer Lisa Sakamoto, Akira Shibanuma, Ekaterina Yarotskaya, Milana Basargina, Mika Kunieda, Masamine Jimba

To enable PROSPERO to focus on COVID-19 registrations during the 2020 pandemic, this registration record was automatically published exactly as submitted. The PROSPERO team has not checked eligibility.

Citation

Rogie Royce Carandang, Jennifer Lisa Sakamoto, Akira Shibanuma, Ekaterina Yarotskaya, Milana Basargina, Mika Kunieda, Masamine Jimba. Roles of Maternal and Child Health Handbook and other Home-based Records on Newborn and Child Health: A Systematic Review. PROSPERO 2020 CRD42020166545 Available from:
https://www.crd.york.ac.uk/prospero/display_record.php?ID=CRD42020166545

Review question

What are the roles of Maternal and Child Health (MCH) Handbook and other home-based records on the promotion of newborn/child health and the prevention and management of newborn/childhood illnesses?

Searches

We will search the following databases: PubMed/MEDLINE, Web of Science, CINAHL, PsycINFO, PsycARTICLES, Academic Search Complete, SocINDEX, Cochrane Central Register of Controlled Trials, DARE, NHS EED, HTA, and Grey Literature (WHO, CDC, ECDC, JICA, UNAIDS, among others). We will also search for Japanese databases: J-STAGE, Ichushi, UTokyo Resource Explorer (TREE). We will hand-search the reference list of articles selected for analysis. We will include all published papers in the English and Japanese language up till January 2020.

Our search strategy will combine both Medical Subject Headings (MeSH) terms and free text terms (in English and Japanese).

Search strategy

https://www.crd.york.ac.uk/PROSPEROFILES/166545_STRATEGY_20200123.pdf

Types of study to be included

We will include original research articles in English and Japanese of all study designs such as randomized controlled trial (RCT), quasi-experimental, cohort, observational, cross-sectional, and other comparative studies as well as multiple case studies and evaluation reports. We will not include single case studies, letters, editorials, reviews, conference abstracts, and books.

Condition or domain being studied

A home-based record is a paper or electronic health record retained and used by women or caregivers in the household to document maternal, newborn, and child health (WHO, 2018). To date, over 163 countries have been using home-based records.

In 1948, the Ministry of Health of Japan introduced the MCH handbook to improve the health of vulnerable mothers and children (Hagiwara, 2013). As of 2016, at least 25 countries used the fully integrated MCH handbook (Osaki 2016).

One study systematically reviews the effectiveness of home-based records on maternal and child health (Magwood, 2019), which was used as a basis for WHO's recommendations on home-based records (WHO, 2018). However, Magwood et al. did not mention about health promotion and management of newborn/childhood illnesses. In addition, they covered only original articles with controlled study designs and written in the English language. In this review, we aim to include original articles in both English and

Japanese language of all study designs. Since Japan is the proponent of the MCH handbook, it would be worthy of including Japanese articles in the analysis. By doing so, we could capture more evidence on the effectiveness of home-based records on newborn/child health.

Participants/population

Participants will include parents, fathers, mothers, and caregivers of children 0-12 years.

Intervention(s), exposure(s)

The intervention of interest is the MCH handbook and other home-based records, available in either hard copy or online, and kept or managed by parents/caregivers.

Comparator(s)/control

The comparator will be no record, conventional information or usual care given to parents/caregivers following childbirth.

Main outcome(s)

Newborn/child health promotion and reporting

Newborn/child health care seeking and care practices

Newborn/childhood illness prevention and management

Newborn/child morbidity and mortality

* Measures of effect

Not applicable

Additional outcome(s)

Parent/caregiver’s health knowledge

Communication within the household and between women/caregivers and health care providers

Satisfaction with services

Continuity of care

* Measures of effect

Not applicable

Data extraction (selection and coding)

Two review authors will be involved in the process of literature search, article screening, and data extraction. The databases will be independently searched using the aforementioned search strategy and identify the studies by title and abstract screening. The team will review the list of articles for eligibility. We will discuss disagreements on the eligibility of study until a consensus is reached. If required, we will consult our supervisor for the final decision.

The data to be extracted include:

title, citation (author, publication year, source), objectives, study design, study setting, study population, sample size, types of home-based records, comparison group, and reported outcomes.

Risk of bias (quality) assessment

We will assess the quality of randomized trials using the risk of bias tools from the Cochrane Handbook. The quality of nonrandomized controlled trials will also be assessed using the same tool. By default, it will receive a judgment of "high risk of bias" for random allocation and allocation concealment. To assess the certainty of the evidence for the included studies, we will apply the GRADE approach. For qualitative studies, we will use the Critical Appraisal Skill Programme (CASP) tool.

Strategy for data synthesis
We will follow the PRISMA checklist for appropriate data synthesis. We will construct a PRISMA flowchart to show the search strategy results at each stage of review. We will conduct a descriptive analysis of individual studies according to the type of intervention, sample size, duration, outcome, quality, and risk of bias. We will analyze the effectiveness of the intervention, based on the nature of reported outcomes. If we find enough studies with quality data, we will conduct a meta-analysis to examine the effectiveness of the MCH handbook and other home-based records on newborn/child health.

Analysis of subgroups or subsets
None

Contact details for further information
Rogie Royce Carandang
rrcarandang@gmail.com

Organisational affiliation of the review
Department of Community and Global Health, The University of Tokyo
http://www.ich.m.u-tokyo.ac.jp/en/index.html

Review team members and their organisational affiliations
Dr Rogie Royce Carandang. Department of Community and Global Health, Graduate School of Medicine, The University of Tokyo, Tokyo, Japan
Assistant/Associate Professor Jennifer Lisa Sakamoto. Department of Community and Global Health, Graduate School of Medicine, The University of Tokyo, Tokyo, Japan
Dr Akira Shibanuma. Department of Community and Global Health, Graduate School of Medicine, The University of Tokyo, Tokyo, Japan
Dr Ekaterina Yarotskaya. Head, Department of International Cooperation, Center for Obstetrics, Gynecology and Perinatology, Ministry of Healthcare, Moscow, Russia
Dr Milana Basargina. Head, Neonatal Department, National Medical Research Center for Children's Health, Ministry of Healthcare, Moscow, Russia
Assistant/Associate Professor Mika Kunieda. Faculty of Policy Management, Keio University Shonan Fujisawa Campus, Tokyo, Japan
Professor Masamine Jimba. Department of Community and Global Health, Graduate School of Medicine, The University of Tokyo, Tokyo, Japan

Type and method of review
Meta-analysis, Systematic review

Anticipated or actual start date
01 February 2020

Anticipated completion date
31 August 2020

Funding sources/sponsors
Department of Community and Global Health, Graduate School of Medicine, The University of Tokyo, Japan

Conflicts of interest

Language
English, Japanese

Country
Japan, Russian Federation

Stage of review
Review Ongoing

Subject index terms status

Subject indexing assigned by CRD

Subject index terms

MeSH headings have not been applied to this record

Date of registration in PROSPERO

28 April 2020

Date of first submission

23 January 2020

Stage of review at time of this submission

Stage	Started	Completed
Preliminary searches	Yes	No
Piloting of the study selection process	Yes	No
Formal screening of search results against eligibility criteria	No	No
Data extraction	No	No
Risk of bias (quality) assessment	No	No
Data analysis	No	No

The record owner confirms that the information they have supplied for this submission is accurate and complete and they understand that deliberate provision of inaccurate information or omission of data may be construed as scientific misconduct.

The record owner confirms that they will update the status of the review when it is completed and will add publication details in due course.

Versions

28 April 2020

PROSPERO
This information has been provided by the named contact for this review. CRD has accepted this information in good faith and registered the review in PROSPERO. The registrant confirms that the information supplied for this submission is accurate and complete. CRD bears no responsibility or liability for the content of this registration record, any associated files or external websites.
