# Supplementary material for: Roles of the Maternal and Child Health Handbook and Other Home-Based Records on Newborn and Child Health: A Systematic Review
Source: Int J Environ Res Public Health. 2021 Jul 13;18(14):7463. doi: 10.3390/ijerph18147463 (PMC8306696; doi:10.3390/ijerph18147463)
Supplement: Supplementary file 1 [file ijerph-18-07463-s001.zip › Text S2. Search strategy.pdf]

Text S2. Search strategy

Strategy 1: Search terms for "newborn"

infant [MeSH] OR infant [tw]
OR newborn [MeSH] OR newborn [tw]
OR neonate [MeSH] OR neonate [tw]

Strategy 2: Search terms for "child"

child [MeSH] OR child [tw]
OR children [MeSH] OR children [tw]

Strategy 3: Search terms for "MCH handbook"

maternal and child health handbook [MeSH] OR maternal and child health handbook [tw]
OR MCH Handbook [MeSH] OR MCH handbook [tw]
OR home-based record [MeSH] OR home-based record [tw]
OR paper-based record [MeSH] OR paper-based record [tw]
OR personal health record [MeSH] OR personal health record [tw]
OR child health record [MeSH] OR child health record [tw]
OR child health book [MeSH] OR child health book [tw]
OR maternal health record [MeSH] OR maternal health record [tw]
OR maternal health book [MeSH] OR maternal health book [tw]
OR maternal and child health book [MeSH] OR maternal and child health book [tw]
OR vaccination record [MeSH] OR vaccination record [tw]
OR vaccination book [MeSH] OR vaccination book [tw]
OR immunization record [MeSH] OR immunization record [tw]
OR immunization book [MeSH] OR immunization book [tw]

Strategy 4: Search terms for "child health promotion and prevention and management of childhood illnesses"

infant health [MeSH] OR infant health [tw]
OR child health [MeSH] OR child health [tw]
OR health of children [MeSH] OR health of children [tw]
OR newborn health [MeSH] OR newborn health [tw]
OR neonate health [MeSH] OR neonate health [tw]
OR health promotion [MeSH] OR health promotion [tw] OR promotion of child health [tw]
OR childhood disease* [tw] OR childhood illness* [tw]
OR disease management [MeSH] OR disease management [tw]
OR pneumonia [MeSH] OR pneumonia [tw] OR respiratory tract disease [MeSH]
OR diarrhea [MeSH] OR diarrhea [tw] OR diarrhoea [tw]
OR tuberculosis [MeSH] OR tuberculosis [tw]
OR fever [MeSH] OR fever [tw]
OR malaria [MeSH] OR malaria [tw]
OR otitis [MeSH] OR ear problems [tw]
OR dengue [MeSH] OR dengue [tw]
OR meningitis [MeSH] OR meningitis [tw]
OR measles [MeSH] OR measles [tw]
OR sepsis [MeSH] OR sepsis [tw] OR septicemia [tw]
OR typhoid fever [MeSH] OR typhoid fever [tw]
OR malnutrition [MeSH] OR malnutrition [tw]
OR HIV [MeSH] OR HIV [tw] OR AIDS [tw]

OR developmental disabilities [MeSH] OR developmental disabilit* [tw] OR developmental disorder* [tw]
OR epilepsy [MeSH] OR epilepsy [tw] OR seizure disorder* [tw]
OR supportive care [tw]
OR treatment [tw] OR therapy [tw]
OR prevention [tw] OR prevention and control [MeSH] OR prevention and control [tw]
OR attitude [MeSH] OR attitude* [tw] OR behavior [MeSH] OR behavior* [tw]
OR practice* [tw]

Combination of search strategies

((infant[MeSH] OR infant[tw] OR newborn[MeSH] OR newborn[tw] OR neonate[MeSH] OR neonate[tw]) AND (child[MeSH] OR child[tw] OR children[MeSH] OR children[tw]) AND (maternal and child health handbook[MeSH] OR maternal and child health handbook[tw] OR MCH Handbook[MeSH] OR MCH handbook[tw] OR home-based record[MeSH] OR home-based record[tw] OR paper-based record[MeSH] OR paper-based record[tw] OR personal health record[MeSH] OR personal health record[tw] OR child health record[MeSH] OR child health record[tw] OR child health book[MeSH] OR child health book[tw] OR maternal health record[MeSH] OR maternal health record[tw] OR maternal health book[MeSH] OR maternal health book[tw] OR maternal and child health book[MeSH] OR maternal and child health book[tw] OR vaccination record[MeSH] OR vaccination record[tw] OR vaccination book[MeSH] OR vaccination book[tw] OR immunization record[MeSH] OR immunization record[tw] OR immunization book[MeSH] OR immunization book[tw]) AND (infant health[MeSH] OR infant health[tw] OR child health[MeSH] OR child health[tw] OR health of children[MeSH] OR health of children[tw] OR newborn health[MeSH] OR newborn health[tw] OR neonate health[MeSH] OR neonate health[tw] OR health promotion[MeSH] OR health promotion[tw] OR promotion of child health[tw] OR childhood disease*[tw] OR childhood illness*[tw] OR disease management[MeSH] OR disease management[tw] OR pneumonia[MeSH] OR pneumonia[tw] OR respiratory tract disease[MeSH] OR diarrhea[MeSH] OR diarrhea[tw] OR diarrhoea[tw] OR tuberculosis[MeSH] OR tuberculosis[tw] OR fever[MeSH] OR fever[tw] OR malaria[MeSH] OR malaria[tw] OR otitis[MeSH] OR ear problems[tw] OR dengue[MeSH] OR dengue[tw] OR meningitis[MeSH] OR meningitis[tw] OR measles[MeSH] OR measles[tw] OR sepsis[MeSH] OR sepsis[tw] OR septicemia[tw] OR typhoid fever[MeSH] OR typhoid fever[tw] OR malnutrition[MeSH] OR malnutrition[tw] OR HIV[MeSH] OR HIV[tw] OR AIDS[tw] OR developmental disabilities[MeSH] OR developmental disabilit*[tw] OR developmental disorder*[tw] OR epilepsy[MeSH] OR epilepsy[tw] OR seizure disorder*[tw] OR supportive care[tw] OR treatment[tw] OR therapy[tw] OR prevention[tw] OR prevention and control[MeSH] OR prevention and control[tw] OR attitude[MeSH] OR attitude*[tw] OR behavior[MeSH] OR behavior*[tw] OR practice*[tw]))

Search strategy for Japanese databases

Ichushi

(母子健康手帳)・原著論文

(母子手帳)・原著論文

J-STAGE

(母子健康手帳)・ジャーナル・査読あり

(母子手帳)・ジャーナル・査読あり
